# Concurrent Chemoradiotherapy Plus Immunotherapy Versus Concurrent Chemoradiotherapy in Locally Advanced Cervical Cancer: A Randomized, Single‐Center, Phase II Trial of Early Tumor Regression and Pro‐Inflammatory Tumor Microenvironment Remodeling

**DOI:** 10.1002/mco2.70856

**Published:** 2026-07-08

**Authors:** Yuhan Sheng, Yu Chang, Yao Jiang, Shujie Wang, Ying Zhou, Minggang Peng, Xiang Kang, Yingchao Zhao

**Affiliations:** ^1^ Cancer Center Hubei Key Laboratory of Precision Radiation Oncology Institute of Radiation Oncology Union Hospital Tongji Medical College Huazhong University of Science and Technology Wuhan Hubei China; ^2^ Key Laboratory of Biological Targeted Therapy Huazhong University of Science and Technology Ministry of Education Wuhan Hubei China; ^3^ Department of Obstetrics and Gynecology Union Hospital Tongji Medical College Huazhong University of Science and Technology Wuhan Hubei China

**Keywords:** cervical cancer, concurrent chemoradiotherapy, immunotherapy, single‐cell RNA sequencing

## Abstract

Concurrent chemoradiotherapy (CCRT) followed by intrauterine brachytherapy is the standard treatment for locally advanced cervical cancer (LACC), yet its efficacy is frequently constrained by an immunosuppressive tumor microenvironment (TME). This randomized, single‐center, Phase II trial (*n* = 18; 2:1 allocation; high‐risk Stages III–IVA LACC) investigated whether adding immunotherapy to CCRT (CICRT) could enhance early tumor regression and overcome immune suppression. In this exploratory analysis, CICRT demonstrated a numerical advantage in tumor reduction over CCRT alone, although the difference did not reach conventional statistical significance (mean residual tumor volume: 3.0% vs. 7.4%, *p* = 0.080, 95% CI: −9.3%–0.6%, *η* ^2^ = 0.180). Single‐cell RNA sequencing of paired biopsies (*n* = 12) revealed that CICRT was associated with pro‐inflammatory remodeling, characterized by the upregulation of MHC‐II and chemotaxis‐related genes in malignant cells and cancer‐associated fibroblasts. Notably, CICRT reduced regulatory T cell niches and exhausted CD8^+^ T cells, alongside a phenotypic shift in tumor‐associated macrophages toward an inflammatory state. These findings demonstrate the potential of CICRT to enhance early tumor regression and mitigate TME suppression, providing an exploratory biological rationale for its therapeutic activity in LACC.

## Graphical abstract

Concurrent chemoradiotherapy plus immunotherapy (CICRT) promotes tumor microenvironment remodeling in locally advanced cervical cancer to enhance antitumor immunity. It reduces regulatory T cells and mitigates CD8^+^ T‐cell exhaustion while reprogramming macrophages toward a pro‐inflammatory phenotype. Furthermore, CICRT upregulates MHC‐II expression and augments cytokine/chemokine secretion in tumor cells and fibroblasts, providing an exploratory biological rationale for its therapeutic activity.

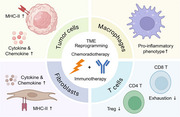

## 1 Introduction

Cervical cancer remains a major global health challenge, with persistent human papillomavirus (HPV) infection identified as the primary etiological factor. In 2020, cervical cancer was ranked as the fourth leading cause of cancer‐related mortality among women worldwide, accounting for approximately 342,000 deaths [1]. Notably, Asia bears a disproportionate burden, representing over 58% of all cases and more than half of the global fatalities. China and India together account for 39% of the global incidence (18% and 21%, respectively) and 40% of total mortality (17% and 23%, respectively) [2]. This significant regional concentration underscores the imperative to optimize therapeutic strategies and improve clinical outcomes for patients in these high‐prevalence areas.

Locally advanced cervical cancer (LACC), classified as Stages IB3–IVA according to the 2018 International Federation of Gynecology and Obstetrics (FIGO) staging system, accounts for 37% (IQR 26%–52%) of all cervical cancer cases, with the highest proportions reported in Asian countries [3]. While cisplatin‐based concurrent chemoradiotherapy (CCRT) followed by brachytherapy is the international standard [3], the 5‐year overall survival (OS) remains unsatisfactory, ranging from 60% to 73% [4, 5]. Various risk factors significantly contribute to in‐field recurrence, including tumor size (> 5 cm), young age (< 40 years), non‐squamous histology, and positive pelvic or para‐aortic lymph nodes (> 1 cm) [6, 7]. Recent evidence has identified tumor volume regression (TVR) during CCRT as a critical prognostic marker [8]. Mayr et al. [9] established that TVR assessed at radiation doses of 40–50 Gy correlates with disease‐free survival (DFS). Further validation by Watanabe et al. [10] showed that a TVR below 72% at Week 5 of radiotherapy is associated with significantly poorer 3‐year DFS and OS. Consequently, the extent of TVR measured following external beam radiotherapy (EBRT) but prior to the initiation of brachytherapy serves as a clinically meaningful surrogate endpoint.

Efforts to improve outcomes in LACC through adjuvant chemotherapy have largely failed [11]. The integration of immune checkpoint inhibitors (ICIs) with CCRT has emerged as a compelling strategy. CCRT induces immunogenic cell death and releases damage‐associated molecular patterns (DAMPs), thereby activating the host immune system [12, 13]. Furthermore, radiotherapy upregulates PD‐L1 expression [14], providing a favorable context for ICIs to enhance CD8^+^ T‐cell activity and modulate regulatory T cells (Tregs). This synergy aims to remodel the immunosuppressive tumor microenvironment (TME), potentially converting “cold” tumors into “hot” ones to maximize efficacy [15].

While several randomized controlled trials (RCTs) have evaluated the combination of ICIs and CCRT in LACC, the results have demonstrated substantial heterogeneity. The KEYNOTE‐A18 study reported that pembrolizumab combined with CCRT significantly prolonged progression‐free survival (PFS) [16], whereas the CALLA trial using durvalumab failed to achieve a significant clinical benefit [17]. These discrepancies may arise from distinct molecular inhibitory mechanisms; specifically, PD‐1 inhibitors like pembrolizumab block interactions with both PD‐L1 and PD‐L2, whereas PD‐L1 blockade preserves the PD‐1/PD‐L2 axis, which may maintain certain immunosuppressive signals in the TME [18, 19], potentially limiting therapeutic efficacy. Furthermore, variation in patient selection and the biological heterogeneity of the TME could influence treatment efficacy. Such inconsistency underscores the necessity of moving beyond clinical endpoints alone to investigate the high‐resolution cellular changes that dictate treatment response or resistance. Recent single‐cell RNA sequencing (scRNA‐seq) studies in cervical cancer have begun to characterize these high‐resolution dynamics, demonstrating that chemoradiotherapy can reprogram malignant cells and macrophage phenotypes [20, 21]. However, most existing scRNA‐seq datasets either focus on standard CCRT alone or are limited by a small sample size [22], leaving the remodeling of the TME under CCRT combined with immunotherapy (CICRT) yet to be fully elucidated.

To address these questions, we utilized tislelizumab, an anti‐PD‐1 antibody that has shown investigative promise in enhancing tumor sensitivity to radiotherapy and chemotherapy across various malignancies, including rectal, pancreatic, and esophageal cancers [23, 24, 25]. We initiated the ChiCTR2200067166 trial to evaluate whether adding tislelizumab to CCRT could enhance early tumor regression in patients with high‐risk LACC. To capture the complex cellular dynamics associated with this combination, we performed scRNA‐seq on paired pre‐ and on‐treatment tumor biopsies. Our findings suggest a numerical trend toward improved early TVR in patients receiving CICRT compared to those receiving standard CCRT. This preliminary clinical signal is accompanied by a more pro‐inflammatory immune microenvironment, as revealed through high‐resolution cellular landscape analysis.

## 2 Results

### 2.1 Patient Characteristics, Safety, and Outcome

**FIGURE 1:**
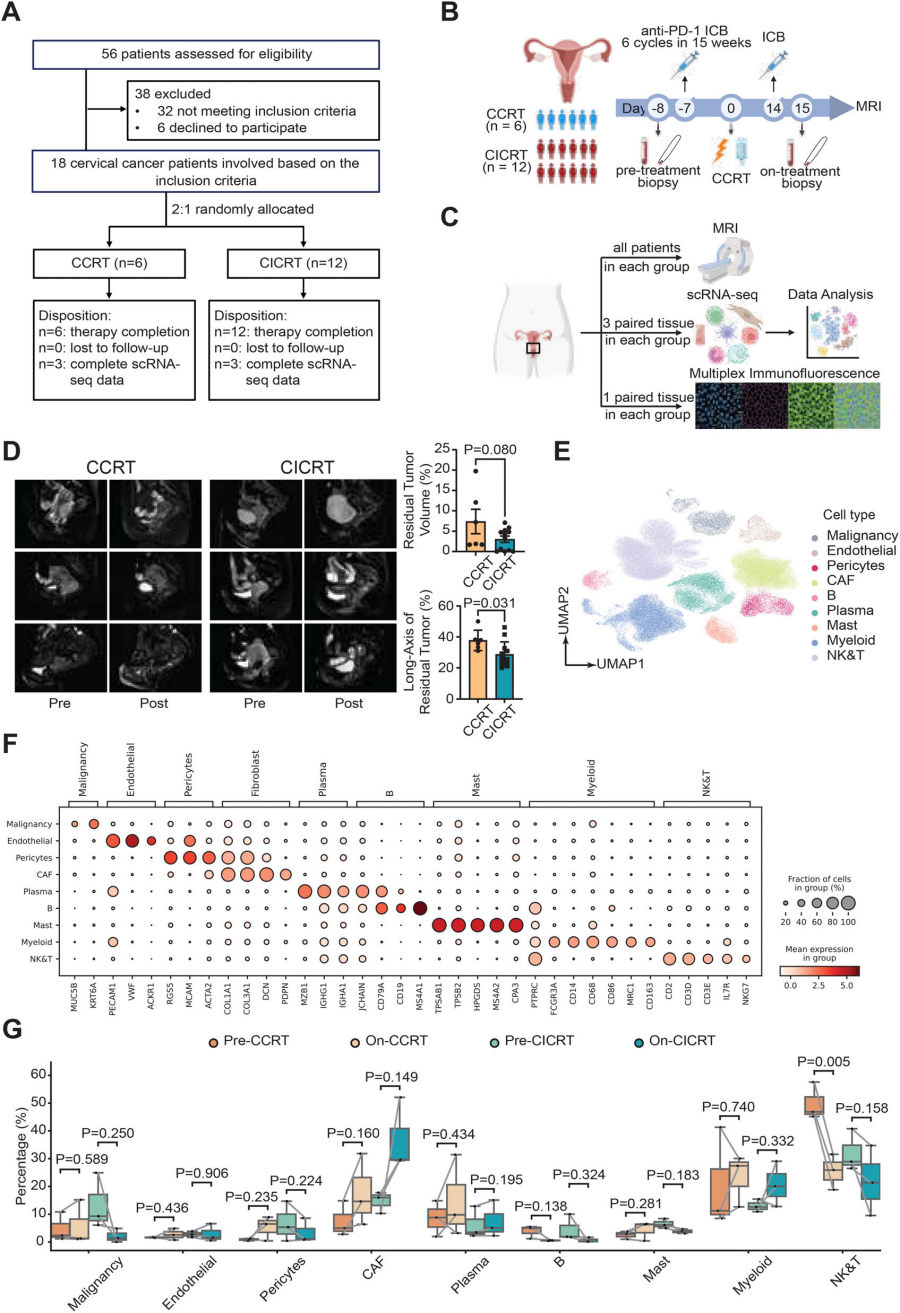
A cellular atlas of concurrent chemoradiotherapy (CCRT) and CCRT plus immunotherapy (CICRT) unveiled through scRNA‐seq. (A) CONSORT diagram of the randomized trial participants and treatment disposition. (B and C) Scheme of the analysis workflow. Figures were created using BioRender (https://www.biorender.com/). (D) Representative MRI images of tumors before and after CCRT and CICRT. Bar graphs (right) show the residual tumor volume and the long‐axis ratio of residual tumor relative to baseline in the CCRT and CICRT groups. *p*‐values were calculated by Student's *t*‐tests. (E) Uniform manifold approximation and projection (UMAP) plot of 80,767 cells from 12 samples (6 paired tumor tissue samples from 6 patients pre‐ and on‐treatment) profiled by scRNA‐seq, colored by cell type. (F) Dot plot showing the expression of canonical marker genes in each cell type. (G) Box plots illustrate the distribution of cell proportions, with each pair of connected dots representing an individual patient. *p*‐values were calculated using paired Student's *t*‐tests.

A total of 18 patients were enrolled in this study, as detailed in the CONSORT diagram (Figure 1A). The median age was 51.5 years (range: 40.3–65.0 years), and the median body mass index (BMI) was 22.3 kg/m^2^ (range: 16.1–32.5 kg/m^2^). Most patients (83.3%) were classified as FIGO Stage IIIC1, followed by Stage IIIC2 (11.1%) and Stage IVA (5.6%). According to the AJCC eighth edition (2018) staging system, the primary tumor (T) stages were T2a (11.1%), T2b (44.4%), T3b (38.9%), and T4 (5.6%). For lymph node involvement, 88.9% and 11.1% of patients were classified as N1 and N2, respectively. There were no significant differences in these baseline characteristics between the CICRT and CCRT groups (Table 1).

**TABLE 1:** Patient demographics and baseline characteristics.

Characteristics	CCRT (*n* = 6)	CICRT (*n* = 12)	Total (*n* = 18)	*p*‐value
Age in years, median (range)	51.9 (40.3–65)	50.7 (41–63.5)	51.5 (40.3–65)	1.000
BMI, median (range)	23.2 (16.1–31.2)	21.9 (17.4–32.5)	22.3 (16.1–32.5)	0.682
FIGO stage, no. (%)				0.687
IIIC1	5 (83.3)	10 (83.3)	15 (83.3)	—
IIIC2	1 (16.7)	1 (8.3)	2 (11.1)	—
IVA	0 (0)	1 (8.3)	1 (5.6)	—
Primary tumor stage (AJCC 2018), no. (%)				0.829
T2a	1 (16.7)	1 (8.3)	2 (11.1)	—
T2b	3 (50)	5 (41.7)	8 (44.4)	—
T3b	2 (33.3)	5 (41.7)	7 (38.9)	—
T4	0 (0)	1 (8.3)	1 (5.6)	—
Lymph node stage (AJCC 2018), no. (%)				1.000
N1	5 (83.3)	11 (91.7)	16 (88.9)	—
N2	1 (16.7)	1 (8.3)	2 (11.1)	—
Performance status, no. (%)				1.000
0	2 (33.3)	5 (41.7)	7 (38.9)	—
1	4 (66.7)	7 (58.3)	11 (61.1)	—
Histology, no. (%)				1.000
Adenocarcinoma	0 (0)	1 (8.3)	1 (5.6)	—
Squamous cell carcinoma	6 (100)	11 (91.7)	17 (94.4)	—

Abbreviation: BMI, body mass index.

Safety outcomes regarding Grade 3 or higher treatment‐related adverse events (TRAEs) are summarized in Table 2. In the CCRT group (*n* = 6), two patients (33.3%) experienced Grade 3 or higher adverse events. Specifically, anemia, diarrhea, nausea, vomiting, urinary tract infection, and decreases in lymphocyte, neutrophil, platelet, and white blood cell counts each occurred in one patient (16.7%). In the CICRT group (*n* = 12), three patients (25.0%) experienced Grade 3 or higher TRAEs. Anemia, nausea, urinary tract infection, and platelet count decrease each occurred in one patient (8.3%), while vomiting and reductions in lymphocyte, neutrophil, and white blood cell counts were observed in two patients each (16.7%).

**TABLE 2:** Grade 3 or higher treatment‐related adverse events.

AE type	CCRT (*n* = 6) No. and (%) of patients Grades 3–5	CICRT (*n* = 12) No. and (%) of patients Grades 3–5
Overall highest grade	2 (33.3)	3 (25.0)
Anemia	1 (16.7)	1 (8.3)
Diarrhea	1 (16.7)	0 (0.0)
Nausea	1 (16.7)	1 (8.3)
Vomiting	1 (16.7)	2 (16.7)
Urinary tract infection	1 (16.7)	1 (8.3)
Lymphocyte count decreased	1 (16.7)	2 (16.7)
Neutrophil count decreased	1 (16.7)	2 (16.7)
Platelet count decreased	1 (16.7)	1 (8.3)
White blood cell decreased	1 (16.7)	2 (16.7)

All 18 patients underwent MRI for tumor volume assessment at the end of pelvic radiotherapy. At a 10% significance level, the CICRT group exhibited a numerical trend toward a greater reduction in residual tumor volume compared to the CCRT group (residual tumor volume 3.0% vs. 7.4%; *p* = 0.080, 95% confidence interval [CI]: −9.3%–0.6%, *η* ^2^ = 0.180); this met the pre‐specified threshold, although it did not reach the conventional 0.05 level. Furthermore, the residual tumor long‐axis ratio relative to baseline was significantly lower in the CICRT group (28.7% vs. 37.7%; *p* = 0.031, 95% CI: −17.2% to −0.9%, *η* ^2^ = 0.259) (Figure 1D and Figure S1B). At a median follow‐up of 19.6 months (range: 8.2–34.2 months), one PFS event occurred in each treatment arm: one patient in the CCRT group progressed at 10 months, and one in the CICRT group at 12 months. All other patients in both arms maintained continuous complete response (CR) throughout the observation period and median PFS was not reached in either group.

### 2.2 Single‐Cell Landscape and Compositional Dynamics of the TME Following CCRT and CICRT

To investigate treatment‐induced alterations in the TME, scRNA‐seq was performed on 12 tumor samples obtained from six patients (*n* = 3 per group), providing paired pre‐treatment and on‐treatment timepoints (Figure 1B,C). In addition, paired specimens from two of these patients (*n* = 1 per group) underwent multiplex immunofluorescence staining (Figure S2). After quality control, a total of 80,767 cells were retained from six paired samples, including 17,013 Pre‐CCRT, 26,722 On‐CCRT, 12,087 Pre‐CICRT, and 24,945 On‐CICRT cells. Following cell clustering and uniform manifold approximation and projection (UMAP)‐based visualization (Figure 1E), major cell lineages were identified using canonical markers: endothelial cells (*PECAM1, VWF, ACKR1*), pericytes (*RGS5, MCAM, ACTA2*), fibroblasts (*COL1A1, COL3A1, DCN*), NK&T cells (*CD2, CD3D, CD3E, NKG7*), myeloid cells (*FCGR3A, CD68*), mast cells (*TPSAB1, TPSB2, MS4A2*), B cells (*CD79A, MS4A1*), and plasma cells (*MZB1, IGHG1*) (Figure 1F). The distribution of cell populations exhibited inter‐sample variability (Figure S3A). The proportion of NK&T cells significantly decreased following CCRT (*p* = 0.005), with a similar but nonsignificant downward trend observed in the CICRT group (*p* = 0.158) (Figure 1G). This observation was further validated using miloR differential abundance analysis, which identified neighborhoods of NK&T cells with significantly reduced abundance following CCRT, whereas no significant changes were detected in the CICRT group (Figure S3C). A decline in peripheral blood lymphocyte counts was observed in both the CCRT and CICRT groups during treatment (Figure S4B). These findings are consistent with previous studies [26] and may be attributed to the high radiosensitivity of T cells [27]. While other cell populations showed no statistically significant shifts, certain trends were noted. In the CICRT group, a consistent decrease in the proportion of malignant cells was observed across all three pairs of samples at the on‐treatment timepoint, although this did not reach statistical significance (Figure 1G). The proportions of cancer‐associated fibroblasts (CAFs) and myeloid cells exhibited a marginal upward trend during treatment in both groups, while B‐cell proportions slightly decreased. Endothelial and plasma cell proportions remained relatively stable throughout the treatment period.

### 2.3 CICRT Is Associated With Enhanced Immune‐Related Gene Expression in Epithelial Cells

**FIGURE 2:**
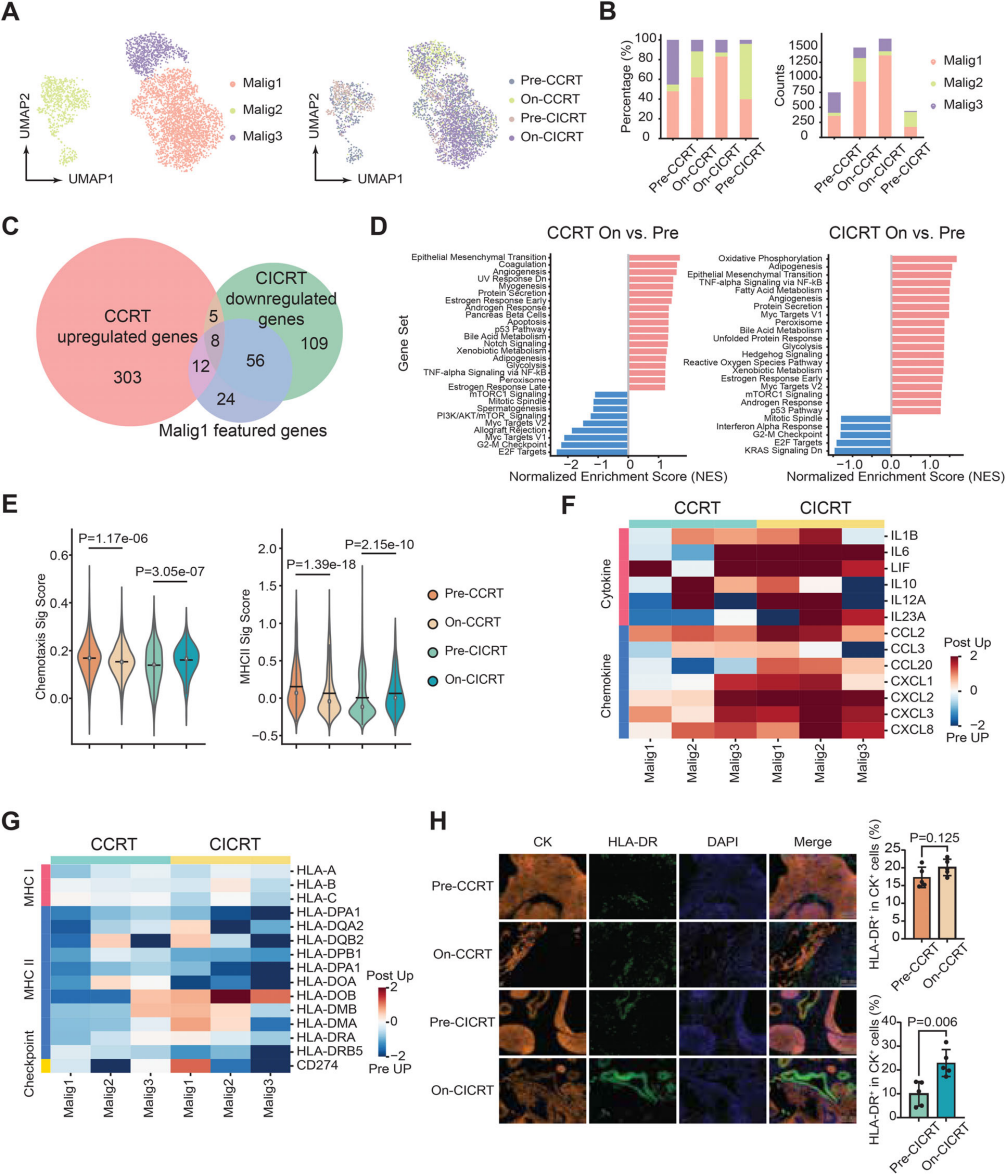
Phenotypic shifts toward an immunostimulatory profile in malignant cells following CICRT. (A) UMAP visualization of 3 malignant cell clusters colored by cluster (left) and condition (right). (B) Distribution and cell numbers of malignant cell subpopulations in each group. (C) Venn diagram showing the overlap of upregulated genes in CCRT, downregulated genes in CICRT, and signature genes of Malig1 cluster. (D) Gene Set Enrichment Analysis (GSEA) of malignant cells: On‐CCRT versus Pre‐CCRT samples (left) and On‐CICRT versus Pre‐CICRT samples (right). (E) Violin plots showing chemotaxis (left) and major histocompatibility complex (MHC) class II (right) signature scores in malignant cells at pre‐ and on‐treatment timepoints (CCRT and CICRT). *p*‐values were determined by a paired two‐sided Wilcoxon signed‐rank test. (F) Fold changes (on‐ vs. pre‐treatment) of cytokine and chemokine gene expression across malignant cell clusters in the CCRT and CICRT groups. Red indicates upregulation, while blue indicates downregulation. (G) Fold changes (on‐ vs. pre‐treatment) of MHC and immune checkpoint gene expression across malignant cell clusters in the CCRT and CICRT groups. Red indicates upregulation, while blue indicates downregulation. (H) Representative immunofluorescence images of CK (orange), HLA‐DR (green), and DAPI (blue) staining in pre‐ and on‐treatment tumor samples from the CCRT and CICRT groups. Merged images highlight the localization of HLA‐DR expression within the CK^+^ epithelial compartment. Bar plots (right) show the quantification of the percentage of HLA‐DR^+^ cells among total CK^+^ epithelial cells. Statistical significance was assessed using paired Student's *t*‐tests based on the quantification of five randomly selected fields per sample.

To further characterize the treatment‐induced molecular shifts within the malignant compartment, we performed reclustering and UMAP analysis specifically on the malignant epithelial cells. These cells were stratified into three transcriptionally distinct subclusters (Figure 2A). Upon treatment, both the CCRT and CICRT groups exhibited a comparable shift in subcluster composition, characterized by an expansion of Malig2 and a contraction of Malig3. The Malig1 subcluster displayed divergent patterns between the two cohorts: its proportion decreased in the CICRT group but increased in the CCRT group (Figure 2B). Consistent with the proportional shifts, differential abundance analysis demonstrated a depletion of Malig1‐associated neighborhoods specifically in the CICRT group (Figure S5B), whereas these neighborhoods remained stable under CCRT. To explore the underlying molecular features, we examined differentially expressed genes (DEGs) between pre‐ and on‐treatment samples (Figure S5C). Notably, a core set of Malig1 subcluster feature genes (Figure 2C)—including *GPRC5A*, *F3*, *MUC4*, *DENND2C*, *S100P*, *MUC1*, *NDRG2*, and *SERPINB2—*was characteristically upregulated following CCRT but markedly suppressed following CICRT. Together, these transcriptomic and abundance patterns support the hypothesis that CICRT may preferentially attenuate the Malig1 subcluster, which otherwise exhibits relative resilience under conventional CCRT.

Gene Set Enrichment Analysis (GSEA) [28] was performed on the DEGs between paired pre‐ and on‐treatment malignant cells within both the CCRT and CICRT cohorts (Figure 2D). In both groups, treatment triggered the upregulation of epithelial–mesenchymal transition (EMT), angiogenesis, p53, and TNF‐α signaling via NF‐κB pathways, while concurrently repressing proliferative pathways such as E2F targets and G2/M checkpoint. Despite these shared responses, changes observed following CCRT were uniquely characterized by the enrichment of pathways involved in coagulation, Notch signaling, and apoptosis, alongside a marked suppression of PI3K/AKT/mTOR signaling and Myc targets. In contrast, CICRT specifically enriched gene sets associated with oxidative phosphorylation, reactive oxygen species (ROS), fatty acid metabolism, and the Hedgehog signaling pathway. Furthermore, while elevated antigen processing has been associated with CCRT in previous studies [20], our analysis revealed that the CICRT group exhibited a more pronounced immune‐related profile of tumor cells, as reflected by higher major histocompatibility complex class II (MHC‐II) and chemotaxis signature scores (Figure 2E). Of note, within the Malig1 subcluster, the CICRT group showed a trend toward upregulation of various cytokines and chemokines, including *IL1B, IL12A, CCL2, CCL3, CCL20, CXCL1, CXCL2, CXCL3*, and *CXCL8* compared to the CCRT group (Figure 2F). This was accompanied by the elevated expression of MHC‐II molecules (*HLA‐DOB, HLA‐DMA*, and *HLA‐DMB*) (Figure 2G), a finding further validated by immunofluorescence staining, which showed a significant increase in HLA‐DR^+^ CK^+^ epithelial cells exclusively in the CICRT group (Figure 2H).

Taken together, these results suggest that CICRT is associated with the upregulation of multiple cytokines and chemokines, accompanied by increased expression of MHC‐II molecules on cancer cells, reflecting a potential shift toward a more immune‐stimulatory TME.

### 2.4 Reprogramming of Myeloid Cells

**FIGURE 3:**
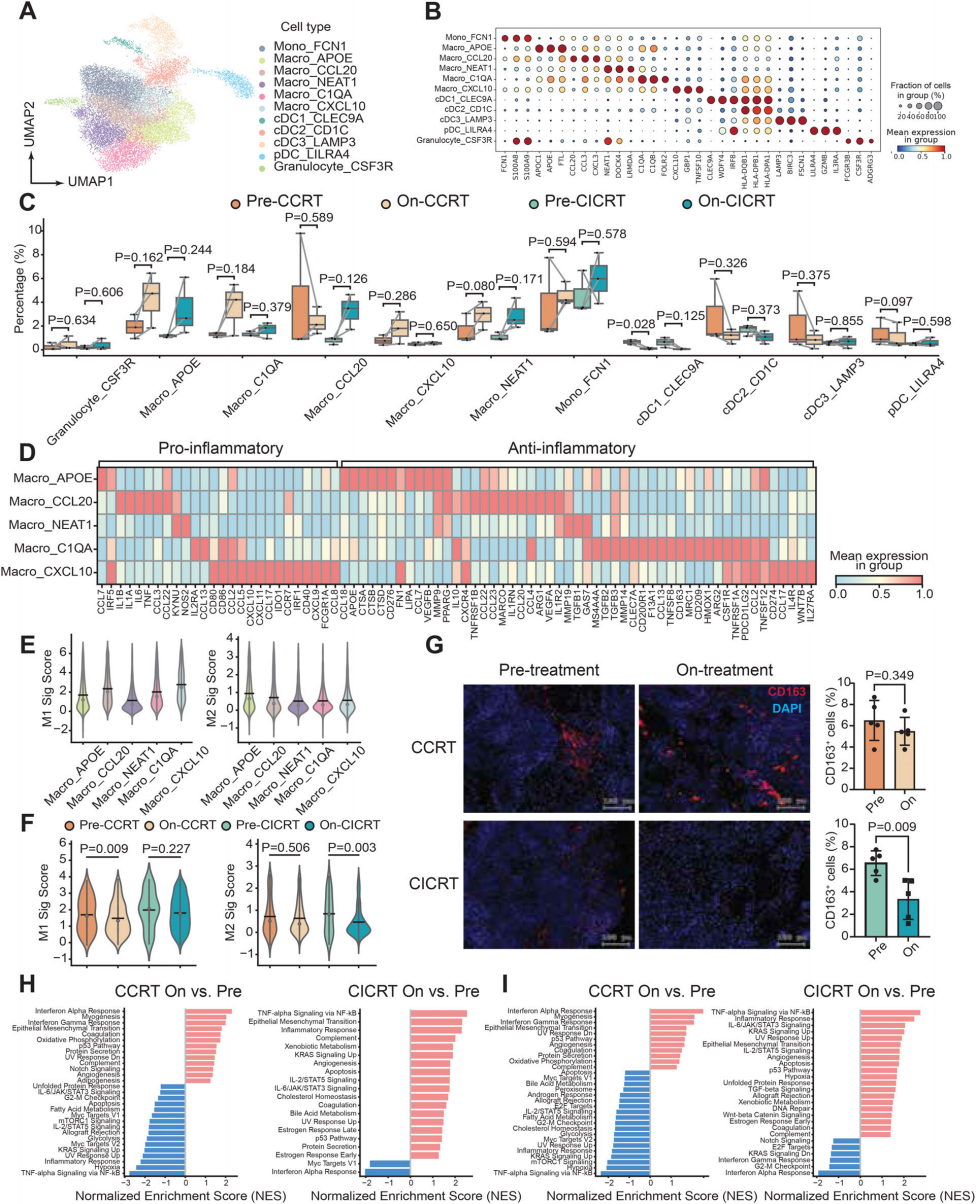
Phenotypic shifts in the myeloid compartment under CICRT and CCRT. (A) UMAP visualization of myeloid clusters colored by cell type. (B) Dot plot showing expression of canonical marker genes in each myeloid subcluster. (C) Box plots showing the abundance of each subcluster within myeloid cells between pre‐ and on‐treatment samples. Each pair of connected dots represents an individual patient. *p*‐values were calculated using paired Student's *t*‐tests. (D) Heatmap depicting the expression of selected pro‐ and anti‐inflammatory genes across the identified macrophage subclusters. (E) Violin plots depicting the M1 signature score (left) and M2 signature score (right) across different macrophage subclusters. (F) Violin plots depicting the M1 (left) and M2 (right) signature scores in Macro_CCL20 cells, comparing pre‐ and on‐treatment timepoints across the CCRT and CICRT cohorts. *p*‐values were determined by a paired two‐sided Wilcoxon signed‐rank test. (G) Representative immunofluorescence staining images of CD163 (red) and DAPI (blue) are shown for pre‐ and on‐treatment samples across the CCRT and CICRT cohorts. Statistical significance was determined by paired Student's *t*‐tests based on the quantification of five randomly selected fields per sample. (H and I) GSEA of Mono/Macro (H) and dendritic cell (DC) clusters (I) comparing on‐ versus pre‐treatment samples within CCRT and CICRT groups.

Myeloid cells are closely associated with the prognosis of cervical cancer patients [29, 30], manifesting both pro‐tumorigenic and anti‐tumorigenic capabilities [31]. Through reclustering analysis, we identified 11 myeloid subclusters (Figure 3A), which were annotated based on their marker genes: one monocyte population (Mono_FCN1); five macrophage populations (Macro_APOE, Macro_CCL20, Macro_NEAT1, Macro_C1QA, Macro_CXCL10); four dendritic cell (DC) populations (cDC1_CLEC9A, cDC2_CD1C, cDC3_LAMP3, pDC_LILRA4); and one granulocyte population (Granulocyte_CSF3R) (Figure 3B). Following treatment, both the CCRT and CICRT cohorts exhibited a downward trend in the proportions of cDC1_CLEC9A and cDC2_CD1C (Figure 3C). Differential abundance analysis confirmed that this reduction was statistically significant within the cellular neighborhoods of these subsets (Figure S6). In contrast, the abundance of cDC3_LAMP3 and pDC_LILRA4 remained stable following CICRT, whereas both populations exhibited a significant neighborhood depletion in the CCRT group (Figure S6). Among the macrophage subsets, Macro_C1QA and Macro_NEAT1 exhibited an enrichment of cellular neighborhoods following CCRT. In contrast, neighborhoods within the Macro_CCL20 subset were specifically expanded following CICRT, while remaining largely stable following CCRT (Figure S6). These findings suggest a treatment‐specific remodeling of the macrophage compartment under CICRT.

All macrophage subclusters demonstrated a heterogeneous signature, encompassing both pro‐ and anti‐inflammatory gene expression (Figure 3D). Among these, Macro_CCL20 exhibited relatively high scores for both M1 and M2 signatures, indicating a mixed polarization state (Figure 3E). We further investigated the treatment‐associated shifts in macrophage polarization states: while the M1 signature score of Macro_CCL20 decreased following CCRT (with no significant change in the M2 score), CICRT maintained a stable M1 score while significantly reducing the M2 signature (Figure 3F). These findings, coupled with the specific neighborhood‐level expansion of this subset, suggest a shift toward a pro‐inflammatory phenotype under CICRT. Immunofluorescence analysis corroborated this by showing that the proportion of CD163^+^ macrophages remained unchanged following CCRT but was significantly reduced in the CICRT group (Figure 3G). Given that CD163^+^ macrophages are typically associated with a pro‐tumorigenic phenotype and poor prognosis in cervical cancer [32], these results suggest that CICRT more effectively remodels the TME, a process characterized by the attenuation of this immunosuppressive population. Furthermore, GSEA revealed distinct immunomodulatory effects across myeloid subclusters. While CCRT primarily enriched the interferon‐α response, CICRT specifically enriched for gene sets related to TNF‐α signaling via NF‐κB, inflammatory response, and IL‐2/STAT5 signaling pathways. IL‐6/JAK/STAT3 signaling was also enriched following CICRT; however, following CCRT, this pathway was downregulated in monocytes and macrophages but remained unchanged in DCs (Figure 3H,I). These findings revealed the divergent regulatory patterns of CCRT and CICRT within the myeloid compartment.

In summary, these findings highlight the dynamic and heterogeneous roles of myeloid cells within the cervical cancer microenvironment, generating the hypothesis that CICRT is associated with a myeloid phenotypic shift toward pro‐inflammatory states—an observational trend further supported by a marked reduction in the infiltration of pro‐tumorigenic CD163^+^ macrophages observed via immunofluorescence.

### 2.5 Reprogramming of T Cells

**FIGURE 4:**
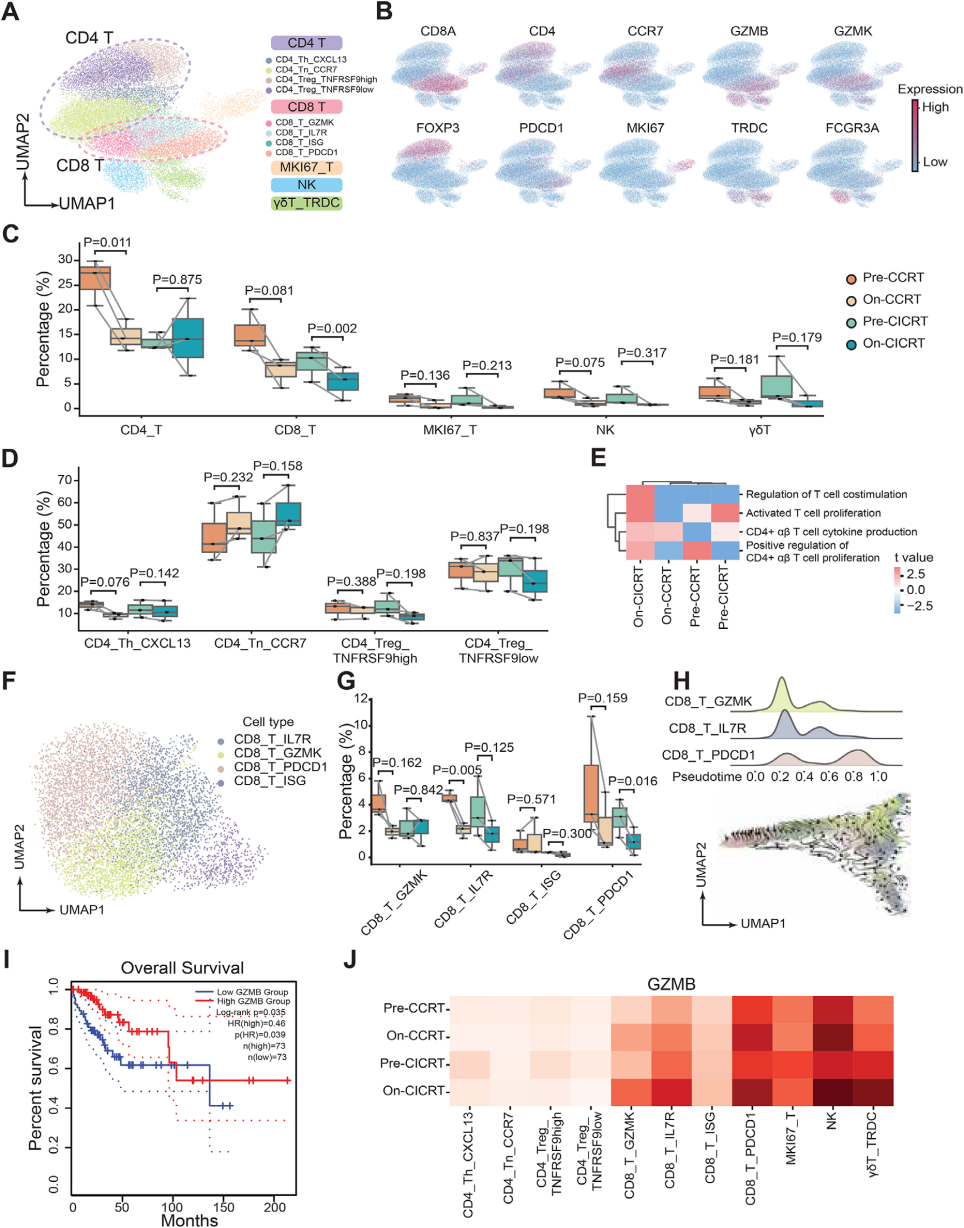
Remodeling of the T‐cell compartment following CCRT and CICRT. (A) UMAP visualization of NK/T‐cell clusters colored by cell type. (B) UMAP plots showing the expression of key markers (*CD8A, CD4, CCR7, GZMB, GZMK, FOXP3*, *PDCD1, MKI67, TRDC*, and *FCGR3A*) used to identify distinct T‐cell subsets, including CD4^+^, CD8^+^, cytotoxic, naive, regulatory, and exhausted T cells. Expression levels are normalized and color‐coded from low (blue) to high (red). (C) Distribution of NK and T‐cell subcluster proportions across treatment groups. *p*‐values were calculated using paired Student's *t*‐tests. (D) Distribution of CD4^+^ T‐cell subcluster proportions across treatment groups. *p*‐values were calculated using paired Student's *t*‐tests. (E) Heatmap illustrating differential pathway activities in CD4^+^ T cells across various conditions. Color intensity reflects *t*‐values derived from a linear model. (F) UMAP visualization of CD8^+^ T‐cell clusters colored by subcluster. (G) Distribution of CD8^+^ T‐cell subcluster proportions across treatment groups. *p*‐values were calculated using paired Student's *t*‐tests. (H) RNA velocity analysis showing a density plot depicting the pseudotime of CD8^+^ T subclusters generated using scVelo (top) and Dynamo (bottom). The bottom plot displays velocity vectors indicating potential differentiation trajectories. (I) Kaplan–Meier survival analysis of overall survival (OS) in TCGA‐CESC patients (*n* = 146). Patients were stratified into High and Low groups based on the top 25% and bottom 25% of *GZMB* expression levels, respectively. Analysis was performed using Gepia2 (http://gepia2.cancer‐pku.cn/#survival), with significance determined by the log‐rank test. (J) Heatmap depicting the expression levels of *GZMB* across NK and T‐cell subclusters under different conditions.

Lymphocytes, especially T cells, are central to effective antitumor immunity [33]. We identified 11 lymphocyte subclusters, comprising four subsets each of CD4_T and CD8_T cells, alongside MKI67_T cells, γδ T cells, and NK cells (Figure 4A,B). The proportions of CD8_T cells and MKI67_T cells exhibited a downward trend following both CCRT and CICRT, suggesting that cytotoxic treatment may lead to a contraction of the T‐cell pool. While the overall population of CD4_T cells significantly declined following CCRT, it remained relatively stable in the CICRT group (Figure 4C). CD4_T cells were further categorized into four groups: two CD4_Treg populations (characterized by *BATF, FOXP3, CTLA4*, and high/low *TNFRSF9* expression, respectively), a CD4_Th_CXCL13 subset (characterized by *CXCL13* and *IFNG*), and a CD4_Tn_CCR7 subset (characterized by *CCR7, IL7R*, and *TCF7*) (Figure S7A,B). CD4_Tn_CCR7 cells exhibited an upward trend in both treatment groups (Figure 4D); differential abundance analysis demonstrated a statistically significant neighborhood‐level expansion specifically in the CICRT group (Figure S7C). Within the Treg cell compartment, both CD4_Treg_TNFRSF9low and CD4_Treg_TNFRSF9high subsets showed only a numerical downward trend (Figure 4D). Notably, differential abundance analysis confirmed a significant reduction at the neighborhood level specifically in the CICRT group (Figure S7C). CD4_T cells in the CICRT group exhibited higher signature scores for the regulation of T‐cell costimulation, activated T‐cell proliferation, and cytokine production compared to the CCRT group (Figure 4E). Immunofluorescence analysis confirmed a significant reduction in the proportion of FOXP3^+^ Tregs within the CD4^+^ population following CICRT (Figure S7D). Collectively, these findings suggest that CICRT is associated with a remodeled CD4^+^ T‐cell compartment. While broad Treg proportions remained stable, neighborhood‐level analysis revealed a localized depletion of Treg‐associated niches alongside the preservation of functional subsets, indicative of a more permissive immune microenvironment.

In terms of CD8_T cells, we identified four distinct subclusters: CD8_T_IL7R (characterized by *IL7R, TCF7*, and *CCR7*), CD8_T_GZMK (characterized by *GZMK*), CD8_T_ISG (characterized by *ISG15, MX1, XAF1*, and *IFI44L*) and CD8_T_PDCD1 (characterized by *CTLA4, HAVCR2*, and *PDCD1*) (Figure 4F and Figure S8A). Analysis of the dynamics of CD8_T cells revealed that CD8_T_IL7R subset decreased in both treatment groups, reaching statistical significance after CCRT. CD8_T_PDCD1 cells also showed a downward trend, with a significant reduction observed exclusively in the CICRT group (Figure 4G). Differential abundance analysis confirmed a significant neighborhood‐level contraction of the CD8_T_PDCD1 and CD8_T_IL7R subsets, alongside a significant expansion of CD8_T_GZMK cells specifically following CICRT. In contrast, no substantial neighborhood‐level shifts were observed in these subsets after CCRT (Figure S8B). To further investigate the differentiation dynamics, we performed RNA velocity analysis using scVelo [34] and Dynamo [35]. CD8_T_GZMK and CD8_T_IL7R clusters were predominantly distributed in early‐to‐intermediate pseudotime stages, indicating their association with less‐differentiated or early effector‐like states. In contrast, CD8_T_PDCD1 cells were enriched at late pseudotime stages, consistent with a terminally exhausted phenotype (Figure 4H). These findings suggest that CICRT is associated with a neighborhood‐level enrichment of early‐stage CD8^+^ T cells and a lower abundance of the terminally exhausted phenotype, reflecting a potentially sustained cytotoxic state. Furthermore, *GZMB* is a key cytotoxic marker and is significantly associated with improved survival in the cervical cancer cohort from The Cancer Genome Atlas (TCGA) (Figure 4I). In our study, *GZMB* exhibited higher expression across multiple NK and T‐cell subsets—including CD8_T_GZMK, CD8_T_PDCD1, NK cells, and γδ T cells—following CICRT compared to CCRT (Figure 4J).

In summary, CICRT was associated with a more immunostimulatory T‐cell landscape, characterized by a neighborhood‐level reduction of Treg cells and a reduced presence of exhausted CD8^+^ T cells, reflecting the preservation of functional T‐cell populations within the TME.

### 2.6 Reprogramming of CAFs

**FIGURE 5:**
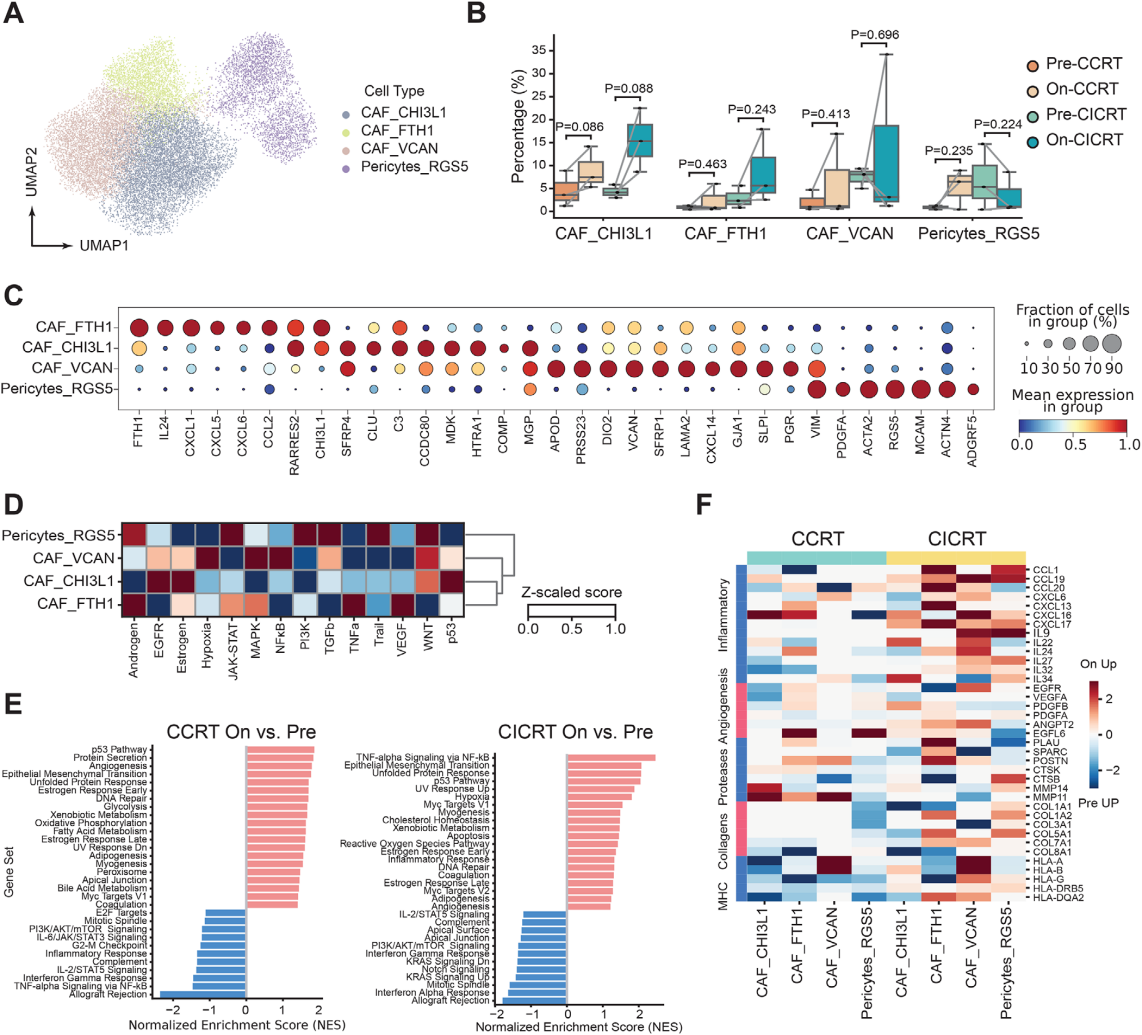
Characterization of cancer‐associated fibroblasts (CAFs) upon CCRT and CICRT. (A) UMAP visualization of CAFs and pericytes colored by cell type. (B) Distribution of pericytes and CAF subcluster proportions across treatment groups. *p*‐values were calculated using paired Student's *t*‐tests. (C) Dot plot showing the expression of feature genes in each CAF subcluster. (D) Heatmap depicting pathway activity across different CAF subtypes in scRNA‐seq data, with pathway activities inferred using PROGENy. (E) GSEA of CAFs comparing on‐ versus pre‐treatment samples within CCRT (left) and CICRT (right) groups. (F) Heatmap depicting the transcriptional remodeling of pericytes and CAF subclusters following treatment. The color scale indicates the relative change in signature gene expression, with red representing upregulation in on‐treatment samples and blue representing higher expression in pre‐treatment samples.

Fibroblasts in the TME play a pivotal role in modulating tumor development and influencing therapeutic responses [36]. To further investigate this population, we reclustered the fibroblasts and identified four distinct subclusters: Pericytes_RGS5 (characterized by *PDGFA, ACTA2, RGS5*), and three populations of CAFs (CAF_CHI3L1, CAF_FTH1, and CAF_VCAN) (Figure 5A,C). Analysis of fibroblast dynamics revealed numerical shifts across subclusters (Figure 5B). Specifically, CAF_CHI3L1 cells exhibited an upward trend following both CCRT and CICRT. In contrast, Pericytes_RGS5 showed a downward trend specifically in the CICRT group. In addition, CAF_FTH1 cells appeared to increase following CICRT but remained relatively stable after CCRT (Figure 5B). Although these proportional shifts did not reach statistical significance, they suggest potential divergent remodeling of the stromal landscape between the two treatment modalities. CAF_FTH1 demonstrated a distinct transcriptional profile characterized by the expression of pro‐inflammatory genes, including *IL24, CXCL1, CXCL5, CXCL6*, and *CCL2*. Consistent with these expression patterns, PROGENy [37] analysis revealed a higher activity score for TNF‐α signaling, a key inflammatory pathway (Figure 5D), underscoring the potential association of the CAF_FTH1 subset with an inflammatory TME. Next, we examined the DEGs of CAFs across both treatment groups (Figure S9) and performed GSEA. Both treatments showed significant enrichment of pathways related to EMT and angiogenesis, highlighting their shared roles in tissue remodeling. CICRT was specifically characterized by the enrichment of inflammatory signaling, including TNF‐α/NF‐κB and the inflammatory response pathways. Notably, these pathways were suppressed in the CCRT group (Figure 5E), suggesting that CAFs under CICRT exhibited a more pronounced pro‐inflammatory and immunostimulatory profile compared to those under CCRT. Consistent with this, we observed a marked upregulation of key inflammatory genes, such as *CCL1, CCL19, CCL20, CXCL6, CXCL17, IL24*, and *IL32* following CICRT. Furthermore, CAFs under CICRT exhibited higher expression levels of collagens and MHC‐II‐related genes compared to those under CCRT (Figure 5F).

In summary, while both treatments are associated with shared patterns of fibroblast remodeling, CICRT is associated with a shift in CAFs toward an immunostimulatory and antigen‐presenting phenotype.

## 3 Discussion

For over two decades, CCRT has remained the cornerstone of treatment for LACC [5, 38]. However, therapeutic resistance and relapse persist as significant challenges, largely driven by the immunosuppressive TME [39, 40]. Recent advances in scRNA‐seq offer unprecedented insights into TME dynamics and the immunological consequences of therapy. In this study, we employed serial tumor sampling alongside scRNA‐seq and multiplex immunofluorescence to systematically characterize the temporal evolution of the TME in LACC patients undergoing CCRT or CICRT. This dynamic sampling strategy helps mitigate the inherent limitations of conventional cross‐sectional studies in capturing treatment‐associated transitions. By delineating the early changes occurring during treatment, our findings provide preliminary insights into the evolving immunomodulatory landscape under the combined pressure of chemoradiotherapy and PD‐1 blockade. Our findings highlight several key features, including the dynamic remodeling of tumor cells, phenotypic shifts in immune subsets, and functional reprogramming of stromal components. These insights provide a preliminary basis for refining personalized therapeutic strategies and improving the long‐term prognosis of patients with LACC.

The optimal timing of ICI initiation in LACC remains a subject of ongoing investigation. A recent study focusing on locally advanced non–small cell lung cancer [41] compared synchronous versus sequential ICI administration, demonstrating that synchronous treatment significantly improved both median PFS (19.0 vs. 12.8 months) and median OS (26.8 vs. 22.2 months), while reducing distant metastasis risk (13.73% vs. 25.53%, *p* = 0.049). Supporting this, subgroup analysis from the PACIFIC trial [42] revealed that initiation of durvalumab within 14 days post‐radiotherapy significantly reduced the risk of disease progression and enhanced the objective response rate (ORR) (34.2% vs. 26.5%) compared to later initiation. Furthermore, the NRG‐GY017 trial demonstrated that patients receiving neoadjuvant and concurrent anti‐PD‐L1 therapy alongside CCRT exhibited a greater expansion of tumor‐associated T‐cell receptor clones compared to concurrent therapy alone, a difference sustained through Day 21 [43]. Mechanistically, radiotherapy serves as a potent immune primer by enhancing tumor antigen presentation, promoting T‐cell activation, and facilitating immune cell homing to irradiated sites, thereby augmenting antitumor immunity [44, 45, 46, 47, 48]. Building on this theoretical framework and clinical evidence, our study adopted a synchronous immunotherapy approach combined with CCRT.

One of our key observations was a trend toward more extensive TVR in the CICRT group compared to the CCRT group. When evaluating treatment response, the inclusion of effect sizes and CIs provides a more statistically grounded perspective than relying solely on *p*‐values. The observed reduction in residual tumor volume under CICRT was characterized by a notable effect size (*η* ^2^ = 0.180), suggesting that the addition of immunotherapy contributes to early tumor reduction. However, the relatively wide 95% CI (−9.3%–0.6%) underscores the inherent precision constraints imposed by limited sample size, reflecting a high level of uncertainty that necessitates validation in larger cohorts. Despite this constraint, the numerical trend toward enhanced early tumor control is aligned with the PFS benefits observed in the KEYNOTE‐A18 study, suggesting that the addition of immunotherapy holds the potential to support rapid tumor control within a relatively short therapeutic window. Currently, multiple clinical studies in LACC suggest that specific subgroups—such as those with > 20% PD‐L1 expression [49], higher baseline levels of PD‐1 on tumor‐infiltrating EMRA CD4^+^ T cells and ICOS‐L on tumor‐associated macrophages (TAMs) [50]—may benefit from immunotherapy combined with CCRT. Our results revealed that the CICRT group was characterized by a prominent reduction in a specific tumor cell subpopulation that remained relatively stable following CCRT. By integrating differential expression analysis with tumor cell dynamics pre‐ and on‐treatment, we identified a shared gene signature comprising *GPRC5A, F3, MUC4, DENND2C, S100P, MUC1, NDRG2*, and *SERPINB2*. Importantly, *GPRC5A* expression is closely associated with reduced immune cell infiltration [51], accelerated cancer cell proliferation [52], and chemoresistance [53]. These findings suggest that this signature warrants further evaluation as a potential candidate for patient stratification in future trials. In addition, we observed a more robust upregulation of MHC‐II‐related molecules and chemotaxis‐associated genes following CICRT than after CCRT, relative to their respective baselines. Interestingly, our results revealed a decline in MHC‐II scores in the CCRT group, which contrasts with prior findings [20] in which brachytherapy was followed by paclitaxel (175 mg/m^2^) and nedaplatin (75–80 mg/m^2^) chemotherapy. This discrepancy likely stems from differences in biopsy timing, radiation delivery, and the specific chemotherapeutic agents utilized.

Regarding the myeloid compartments, monocytes and macrophages underwent complex remodeling following treatment. FCN1^+^ myeloid cells, which have been reported to drive inflammation [54] and correlate with prognosis in multiple cancers [55], remained relatively stable in our cohort. In contrast, we observed a consistent increase in Macro_CCL20 following CICRT across the cohort. While this subset initially exhibited high scores for both M1 and M2 signatures, CICRT led to a reduction in the M2 score while maintaining a stable M1 signature. This suggests a phenotypic shift in these macrophages characterized by the attenuation of their immunosuppressive properties. Crucially, the immunosuppressive CD163^+^ macrophages, typically markers of poor prognosis and relapse [56], were significantly reduced following CICRT. These preliminary findings provide support for the potential association of CICRT with a shift in the myeloid microenvironment toward a more immunostimulatory state.

Our findings provide important insights into the T‐cell dynamics within the TME following CICRT, highlighting a rearranged T‐cell landscape compared to CCRT. While overall Treg proportions showed no significant changes from pre‐ to on‐treatment timepoints, neighborhood‐level analysis revealed a localized depletion of Treg‐associated niches during CICRT, pointing to a potential trend toward a less immunosuppressive milieu. Previous research has indicated that while radiotherapy increases CD8^+^ T‐cell infiltration, these cells often transition toward terminal exhaustion [57]. Given that a higher proportion of progenitor exhausted T cells has been linked to durable clinical benefits [58], the composition of the exhausted T‐cell pool remains a critical factor. One of the most striking T‐cell dynamics observed in our study was the statistically significant reduction in CD8_T_PDCD1 cells specifically within the CICRT group. This observed contraction of terminally exhausted cells coincided with a potential restructuring of the T‐cell pool toward a more functional, effector‐like landscape.

CAFs represent a highly heterogeneous population known to play pivotal roles in shaping the TME by promoting angiogenesis, remodeling the extracellular matrix, inducing drug resistance, and modulating immune responses [59]. While radiotherapy has been shown to significantly alter the CAF phenotype, leading to both pro‐tumorigenic and anti‐tumorigenic effects [60, 61], our study highlights distinct fibroblast responses under CICRT versus CCRT. Specifically, CAFs in the CICRT group exhibited a robust enrichment of pro‐inflammatory signaling pathways, most notably TNF‐α and NF‐κB signaling, which were conversely suppressed following CCRT. This was accompanied by the elevated expression of several cytokines and chemokines, suggesting that CICRT may favor a more immunogenic stromal environment. Furthermore, the upregulation of MHC‐II‐related molecules in CAFs following CICRT suggests an enhanced antigen‐presenting profile at the transcriptional level. While its direct functional role in facilitating enhanced T‐cell activation and subsequent immune surveillance remains to be experimentally validated, this transcriptional shift closely aligns with broader observational trends toward a more immunogenic TME.

Ample evidence suggests that radiotherapy acts as a biological catalyst, fostering an “in situ” vaccine effect by liberating tumor‐associated antigens during cell death, which subsequently triggers a systemic immune response. A hallmark of this phenomenon is the abscopal effect, characterized by the paradoxical shrinkage of distant, non‐targeted lesions following localized irradiation, highlighting the capacity of radiotherapy to transform a local intervention into a systemic therapeutic response. However, the immunomodulatory impact of radiotherapy is a double‐edged sword. While it primes the immune system, radiation can simultaneously trigger compensatory immunosuppressive mechanisms within the TME. These include the recruitment of Tregs and myeloid‐derived suppressor cells [62], which may ultimately blunt the efficacy of the antitumor response. To distinguish whether our observations reflect specific therapeutic synergy or nonspecific bystander phenomena, we delineated the longitudinal evolutionary trajectories of the CICRT and CCRT cohorts and identified distinct patterns of TME remodeling: while CCRT may induce a baseline inflammatory response, the CICRT group was characterized by a more coordinated pro‐inflammatory shift, marked by a neighborhood‐level depletion of Treg niches alongside fewer exhausted CD8^+^ T cells, and the pro‐inflammatory remodeling of tumor and myeloid compartments. This suggests that while radiotherapy initiates an immune flux, immunotherapy could potentially support this response toward a more productive and sustained antitumor landscape. Collectively, these insights into the complementary remodeling capacities of CICRT provide a valuable foundation for optimizing combination strategies in cervical cancer.

## 4 Limitations of the Study

This study has several limitations that should be acknowledged. First, as a Phase II trial with a modest sample size, the statistical power to detect differences across subgroups was restricted. While we observed a numerical trend, these results should be interpreted as exploratory signals rather than definitive therapeutic outcomes. Larger, multi‐center randomized studies are warranted to validate these findings. Second, while scRNA‐seq was utilized to dissect the TME dynamics, the constrained number of paired biopsies may not fully account for the substantial inter‐patient heterogeneity. Due to the scant clinical biopsy material, these findings lack direct functional validation in experimental models, and they should thus be interpreted with caution as hypothesis‐generating, requiring further validation in expanded cohorts and functional studies. Third, the study population was relatively homogeneous, which may limit the generalizability of our findings to more diverse cohorts across different geographic and ethnic backgrounds. In addition, due to the exploratory nature of this study, certain comprehensive analyses—such as genomic correlations with treatment response—were not systematically performed. Finally, the relatively short follow‐up duration (median: 19.6 months) and the small number of survival events (one progressive disease event in each arm) preclude definitive conclusions regarding long‐term PFS benefit or the ultimate durability of the observed therapeutic responses.

## 5 Conclusions

This study suggests that integrating immunotherapy into standard treatment has the potential to support early tumor reduction and reshape the immunosuppressive TME in high‐risk LACC patients. By analyzing tumor tissues collected during the early course of treatment—prior to conventional radiographic assessment—we were able to delineate preliminary dynamic changes within the immune landscape. The observed numerical trends in tumor reduction, coupled with the evidence of TME remodeling, offer valuable insights into how immunotherapy may facilitate local control and provide a preliminary biological rationale for combination therapeutic strategies in LACC. These findings highlight the value of incorporating translational endpoints, such as immune cell dynamics during chemoradiotherapy, into future clinical studies.

## 6 Methods

### 6.1 Study Population

This randomized, Phase II trial was conducted at Union Hospital, Tongji Medical College, Huazhong University of Science and Technology (Wuhan, China). Eligible participants were aged 18 years or older and had newly diagnosed, high‐risk LACC (squamous or adenocarcinoma), classified as FIGO 2018 Stages III–IVA. To ensure reliable tissue acquisition for both pre‐treatment evaluation and on‐treatment biomarker studies, we established a minimum cervical tumor diameter of ≥ 5 cm as an inclusion criterion. In addition, all enrolled patients were required to have lymph nodes with a short‐axis diameter of ≥ 1 cm on pre‐treatment imaging. Eligible patients were also required to have an Eastern Cooperative Oncology Group (ECOG) performance status score of 0 or 1 [63], a tumor tissue sample obtained by core, incisional, or excisional biopsy, and adequate organ function. The research opened on September 6, 2022. The first patient was enrolled on September 15, 2022, and the last patient was enrolled on November 18, 2024.

### 6.2 Study Design and Treatments

This study was an open‐label, randomized (2:1), prospective, Phase II trial with two experimental arms. Patients in the CICRT group received six cycles of tislelizumab (200 mg every 3 weeks), beginning 7 days before and continuing concurrently with CCRT (Design schema shown in Figure 1B). The CCRT regimen included 3–5 weekly cycles of cisplatin (40 mg/m^2^), combined with pelvic EBRT (50.4 Gy in 28 fractions), including a boost of 6–15 Gy to involved lymph nodes, followed by brachytherapy (28–35 Gy in 4–5 fractions). Radiotherapy quality was centrally reviewed before study initiation and continuously monitored for each patient's treatment plan.

Tumor biopsies were obtained 1 week before and 2 weeks after the initiation of CCRT. MRI scans were performed within 2 weeks prior to treatment and 1 week post‐CCRT completion. MRI data were imported into the Eclipse planning system, where an oncologist and a radiologist independently delineated gross tumor volume of the primary tumor (GTVp) on sagittal T2‐weighted images (Figure S1A). The final GTVp was calculated as the mean value of both assessments.

The primary objective of this study was to evaluate early tumor regression after CCRT, measured by the percentage of residual tumor volume at 1‐week post‐CCRT. In light of the exploratory nature of this study, an effect size of Cohen's *d* = 1.2 was assumed, indicating a mean difference between groups of approximately 1.2 times the standard deviation. Based on a two‐sided significance level of *α* = 0.10, a sample size of 18 patients (6 in the CCRT group and 12 in the CICRT group) would provide approximately 74% statistical power to preliminarily assess potential differences between groups. Patients were randomly assigned using the minimization method with a random component. Randomization was stratified by FIGO Stage (IIIC1 vs. non‐IIIC1) and primary tumor T Stage (T2a/T2b vs. T3b/T4), to ensure balanced distribution of these prognostic factors.

Secondary endpoints included safety and tolerability, evaluated by the incidence of grade ≥ 3 TRAEs graded according to the Common Terminology Criteria for Adverse Events (CTCAE) version 5.0, and PFS, defined as the time from treatment initiation to either radiographic disease progression or death from any cause.

### 6.3 Single‐Cell Library Construction and Sequencing

Fresh tumor tissue was dissociated into single‐cell suspension, stained with 0.4% trypan blue, and assessed under a microscope. Samples with > 80% viability were used for downstream processing. Libraries were prepared using the DNBelab C Series High‐throughput Single‐cell RNA Library Preparation Set V3.0 (MGI, China) according to the manufacturer's instructions. Oligo and cDNA libraries were indexed, circularized, and subjected to rolling circle amplification to generate DNA nanoballs (DNBs), which were sequenced using combinatorial Probe‐Anchor Synthesis (cPAS) on the MGI sequencing platform.

### 6.4 Single‐Cell RNA‐seq Data Analysis

Downstream analysis was performed using Scanpy (v1.9.6) and Seurat (v5.0.1). In the quality control step, cells were retained if they had more than 200 genes, unique molecular identifier counts ranging from 10,000 to 70,000, less than 0.2% hemoglobin‐related gene counts, and less than 15% mitochondrial gene counts. Doublets were predicted using Scrublet and removed. After normalizing and log‐transformation, 6000 highly variable genes were selected followed by principal component analysis and batch effects removal from samples using Harmony. UMAP and Leiden were utilized for dimension reduction and clustering, respectively. Cancer cells were identified by copy number instability, which was assessed with the R package inferCNV. Cell types were annotated through assessment of canonical and cluster‐specific markers. Cells with more than two canonical cell‐type markers were excluded. The FindMarkers function was used to identify DEGs between conditions using the Wilcoxon rank‐sum test, with parameters set to logfc.threshold = 0.25 and min.pct = 0.1. Genes with an adjusted *p*‐value < 0.05 and |log2 fold change| > 0.25 were considered significant. Signature gene sets were generated based on MSigDB, and the signature score was calculated using the sc.tl.score function, unless otherwise specified. To identify cell state abundance shifts between groups, we performed differential abundance analysis using the miloR [64], which utilizes a *k*‐nearest neighbor (KNN) graph‐based approach to detect localized changes in cell states.

### 6.5 Multiplex Immunofluorescence

Multiplex immunofluorescence was conducted using the Opal 7‐color Manual IHC Kit (NEL801001KT, PerkinElmer). Tissue samples were fixed in formalin, paraffin‐embedded, and sectioned. Sections were rehydration through a graded ethanol series (100%, 90%, 80%, and 70%), 5 min each. Antigen retrieval involved immersing slides in 10 mM citrate buffer (pH 6.0) and microwaving for 3 min, followed by cooling at room temperature for 15–20 min. Slides were blocked for 10 min at room temperature, then incubated overnight at 4°C with primary antibodies (Table S1), followed by a 10‐min room temperature incubation with secondary antibodies. Opal staining was then applied for 10 min at room temperature. Antigen retrieval was repeated to remove antibodies, followed by TBST washing, and antibody incubations were repeated until all markers were labeled. Nuclear visualization involved DAPI staining for 10 min, followed by TBST washing and mounting (VECTASHIELD HardSet Antifade Mounting Medium, H‐1400, Vector Labs). The imaging was performed using the automated quantitative pathology imaging system (TissueFAXS SpectraS, Tissue Gnostics). For quantification, five randomly selected high‐power fields were acquired per tissue section, and the data were used for downstream statistical analyses.

### 6.6 Statistical Analysis

Continuous variables were summarized by median and range and compared using the Wilcoxon rank‐sum test or Student's *t*‐test, while categorical variables were presented as numbers and percentages and analyzed using either the chi‐square test or Fisher's exact test. Cox proportional hazards models were utilized to compute hazard ratios (HRs) and 95% CIs. Differences between pre‐ and on‐treatment groups were analyzed using paired Student's *t*‐test.

## Author Contributions

Y.S., Y.C., and Y.J. conducted experiments and clinical trial, analyzed data, and drafted original manuscript. S.W. and Y.Zhou contributed to data collection. Y.Zhao, M.P., and X.K. supervised the study, participated in trial design and execution, and critically reviewed and edited the manuscript. All authors have read and approved the final manuscript.

## Funding

This work was supported by the National Natural Science Foundation of China (Grant No. 82102843 and 82473258) and the Natural Science Foundation of Hubei Province, China (Grant No. 2023AFB1074).

## Ethics Statement

This study was registered in the China Clinical Trial Registry (ChiCTR2200067166) and approved by the Medical Ethics Committee of Tongji Medical College, Huazhong University of Science and Technology (Ethics approval number: [2022] Lun‐Shen‐Zi No. 0642). It was conducted in accordance with the Declaration of Helsinki and all applicable regulatory requirements. Written informed consent was obtained from all participants.

## Conflicts of Interest

The authors declare no conflicts of interest.

## Supporting information




**Supporting File: 1** mco270856‐sup‐0001‐SuppMat.docx

## Data Availability

The single‐cell RNA sequencing data generated in this study have been deposited in the China National Center for Bioinformation/Beijing Institute of Genomics, Chinese Academy of Sciences (CNCB‐NGDC) under accession number PRJCA058644. Additional data and the code of this study can be obtained by contacting the corresponding author upon reasonable request. TCGA data used in this study were analyzed before January 2025.
