# Supplementary material for: Concurrent Chemoradiotherapy Plus Immunotherapy Versus Concurrent Chemoradiotherapy in Locally Advanced Cervical Cancer: A Randomized, Single‐Center, Phase II Trial of Early Tumor Regression and Pro‐Inflammatory Tumor Microenvironment Remodeling
Source: MedComm (2020). 2026 Jul 8;7(7):e70856. doi: 10.1002/mco2.70856 (PMC13346770; doi:10.1002/mco2.70856)
Supplement: Supplementary file 1 — Supporting File: 1 mco270856‐sup‐0001‐SuppMat.docx [file MCO2-7-e70856-s001.docx]

**Supplementary Information**

**Concurrent Chemoradiotherapy Plus Immunotherapy vs Concurrent Chemoradiotherapy in Locally Advanced Cervical Cancer: A Randomized, Single-Center, Phase II Trial of Early Tumor Regression and Pro-inflammatory Tumor Microenvironment Remodeling**

Yuhan Sheng^1,2#^, Yu Chang^1,2#^, Yao Jiang^1,2^, Shujie Wang^1,2^, Ying Zhou^1,2^, Minggang Peng^3*^, Xiang Kang^3*^, Yingchao Zhao^1,2*^

**Affiliations**

^1^Cancer Center, Hubei Key Laboratory of Precision Radiation Oncology, Institute of Radiation Oncology, Union Hospital, Tongji Medical College, Huazhong University of Science and Technology, Wuhan, Hubei 430022, China.

^2^Key Laboratory of Biological Targeted Therapy, Huazhong University of Science and Technology, Ministry of Education, Wuhan, Hubei 430022, China.

^3^Department of Obstetrics and Gynecology, Union Hospital, Tongji Medical College, Huazhong University of Science and Technology, Wuhan, Hubei 430022, China.

***Corresponding author**

Minggang Peng, Email: [mgpengwh@163.com](mailto:mgpengwh@163.com)

Xiang Kang, Email: [kchigo@163.com](mailto:kchigo@163.com)

Yingchao Zhao, Email: [2006xh0836@hust.edu.cn](mailto:2006xh0836@hust.edu.cn) (Lead Contact)

Address: Cancer Center, Union Hospital, Tongji Medical College, Huazhong University of Science and Technology, Wuhan 430022, China.

^#^These authors contributed equally to this article.

The authors have declared that no conflict of interest exists.

**Running Head**: CICRT-Induced TME Remodeling in LACC


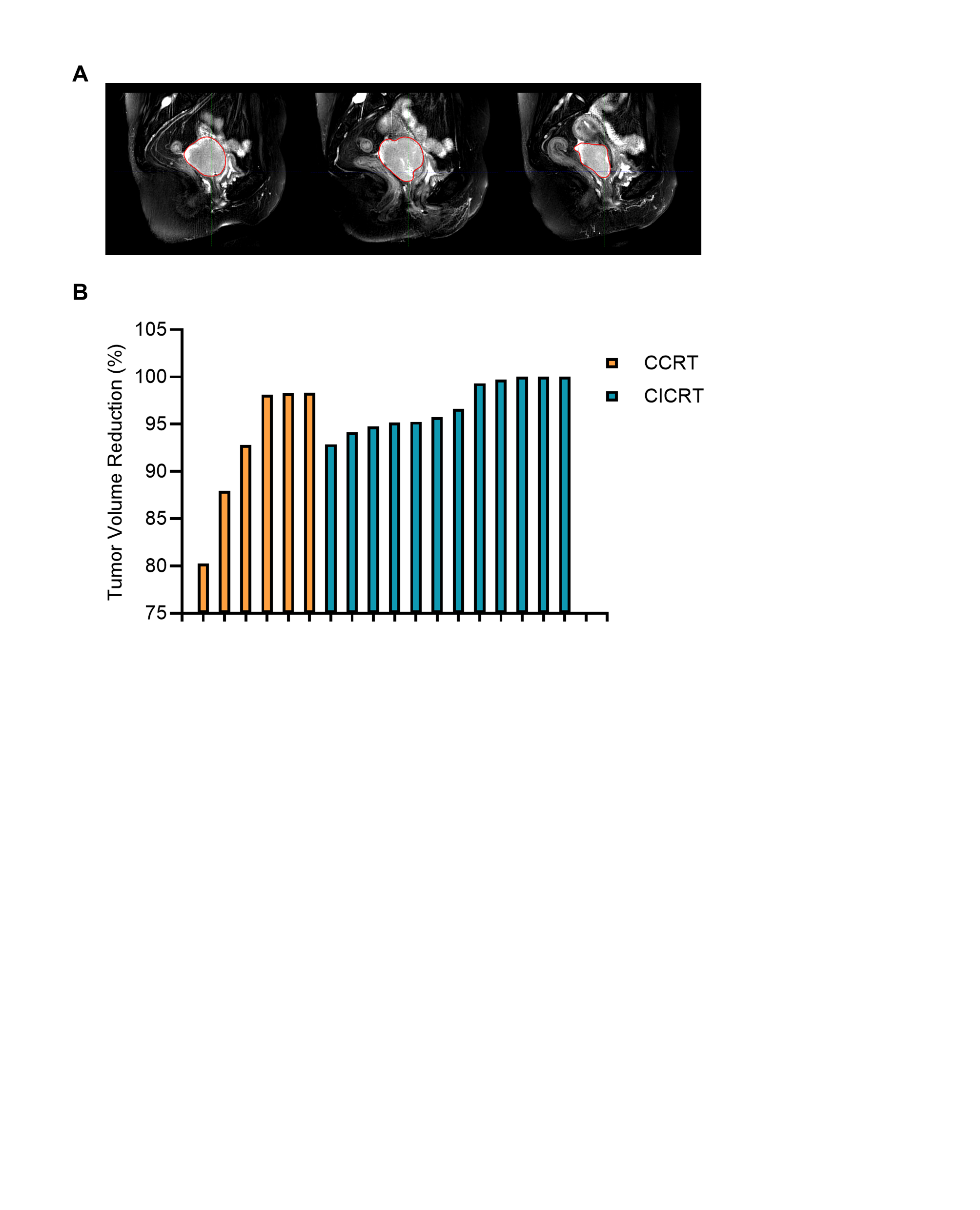
 **Figure S1. Radiologic assessment of tumor regression following treatment.**

**A.** Representative MRI images of a patient were imported into the Eclipse planning system, where an experienced radiation oncologist and a radiologist independently delineated the tumor volume on the sagittal T2-weighted images, defining it as GTVp. The average volume obtained from both specialists was taken as the final GTVp.

**B.** Waterfall plot showing tumor size reduction in individual patients after CCRT (yellow) and CICRT (blue). Each bar represents an individual patient.


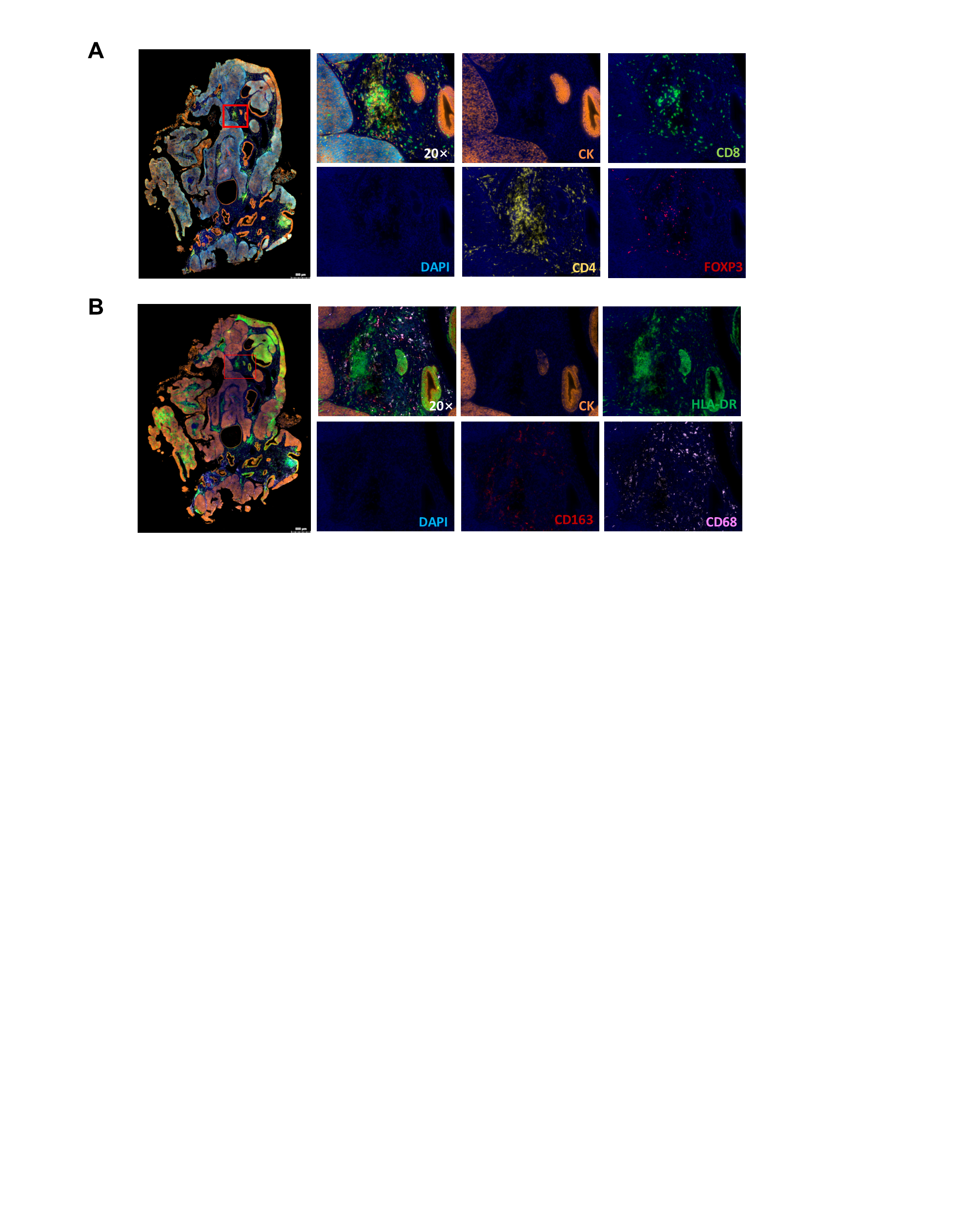
**Figure S2. Representative multiplex immunofluorescence staining of two antibody panels in locally advanced cervical cancer (LACC) tissue.**

**A-B.** Serial sections of the same tumor tissue stained with Panel 1 (A) and Panel 2 (B). The leftmost images display low-magnification overviews with regions of interest highlighted (red boxes). Corresponding higher-magnification (20×) images show staining for DAPI (blue), CK (tumor cells), and selected immune markers. Panel 1 (A) targets CD4, CD8, and FOXP3, while Panel 2 (B) targets HLA-DR, CD163, and CD68.

**
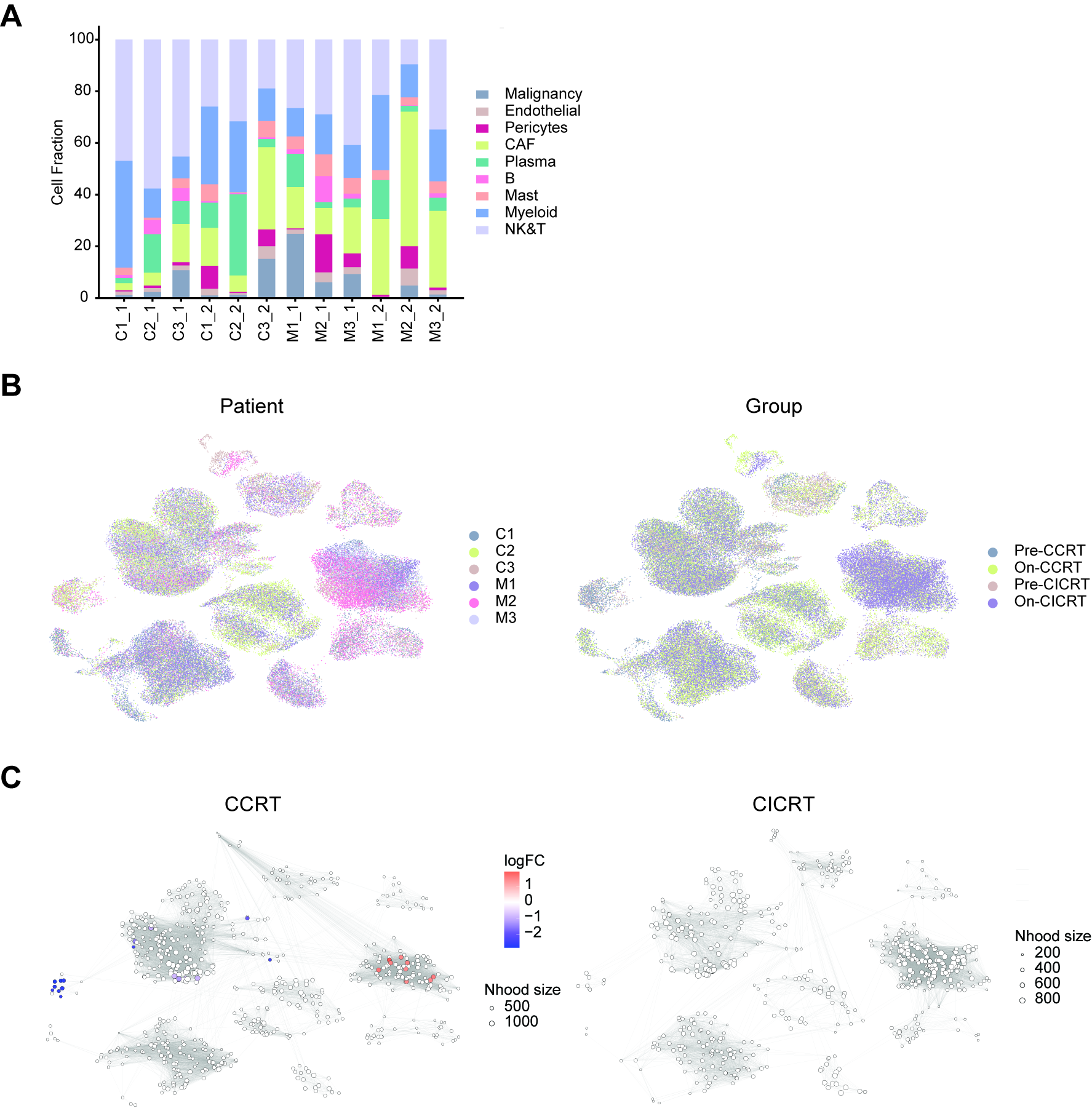
**

**Figure S3. Single-cell landscape and differential cellular abundance of the tumor microenvironment (TME) following treatment.**

**A.** Distribution of cell populations in individual samples.

**B.** UMAP plot of cells from 12 samples (6 paired tumor tissue samples from 6 patients pre- and on-treatment) profiled by scRNA-seq, colored by patient (left) and condition (right).

**C.** Differential abundance analysis of cell neighborhoods. Milo graphs illustrating the shift in cellular abundance between pre- and on-treatment samples for the CCRT (left) and CICRT (right) groups. Each node represents a cell neighborhood, with color intensity indicating the log2-fold change (log2FC). Red denotes enrichment in on-treatment samples, while purple denotes depletion. Node size reflects the number of cells per neighborhood (Nhood size).

**
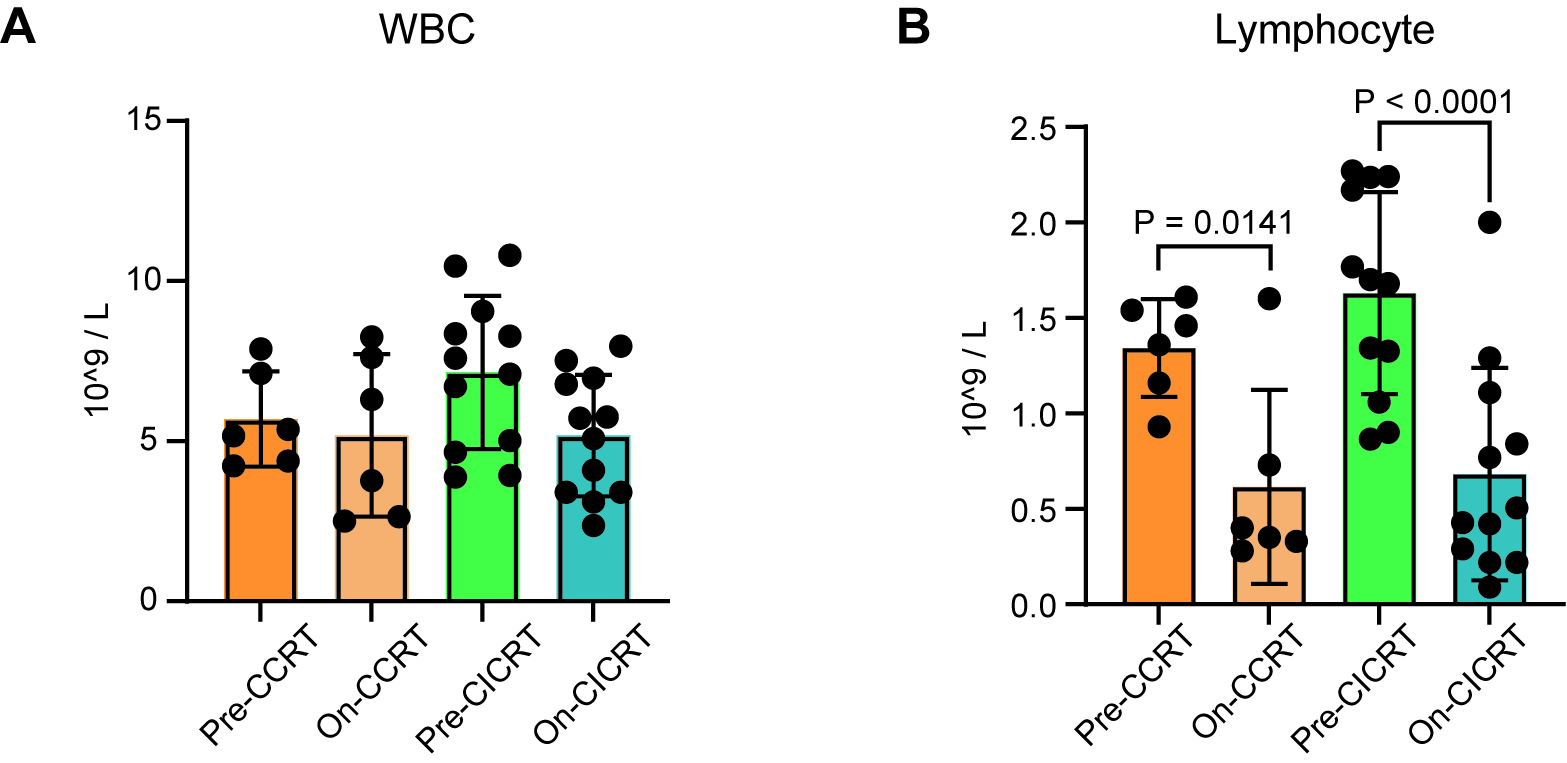
**

**Figure S4. Longitudinal changes in peripheral white blood cell and lymphocyte counts during treatment.**

**A.** Peripheral blood white blood cell (WBC) counts at pre- and on-treatment timepoints. *p*-values were calculated using paired Student’s *t*-tests.

**B.** Peripheral blood lymphocyte counts at pre- and on-treatment timepoints. *p*-values were calculated using paired Student’s *t*-tests.


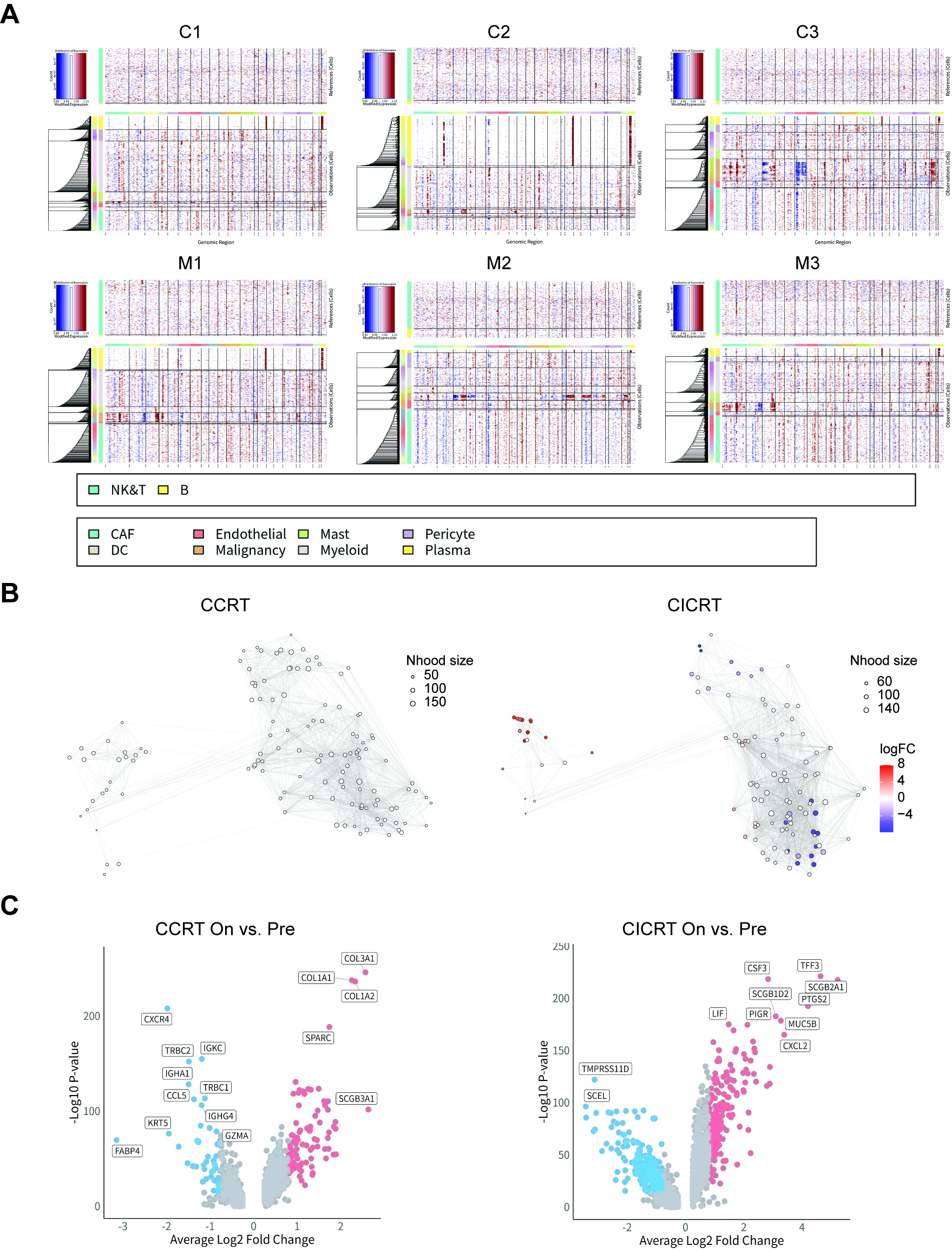


**Figure S5.** **Treatment-induced remodeling of the malignant cell landscape and gene expression.**

**A.** Inferred CNV profiles of malignant cells across individual patients derived from scRNA-seq data.

**B.** Differential abundance analysis of tumor cell neighborhoods. Milo graphs illustrating the shift in cellular abundance between pre- and on-treatment samples for the CCRT (left) and CICRT (right) groups. Each node represents a cell neighborhood, with color intensity indicating the log2-fold change (log2FC). Red denotes enrichment in on-treatment samples, while purple denotes depletion. Node size reflects the number of cells per neighborhood (Nhood size).

**C.** Volcano plots depicting the differential gene expression in malignant cells between pre- and on-treatment samples for the CCRT (left) and CICRT (right) groups. Colored points highlight significantly differentially expressed genes (DEGs).


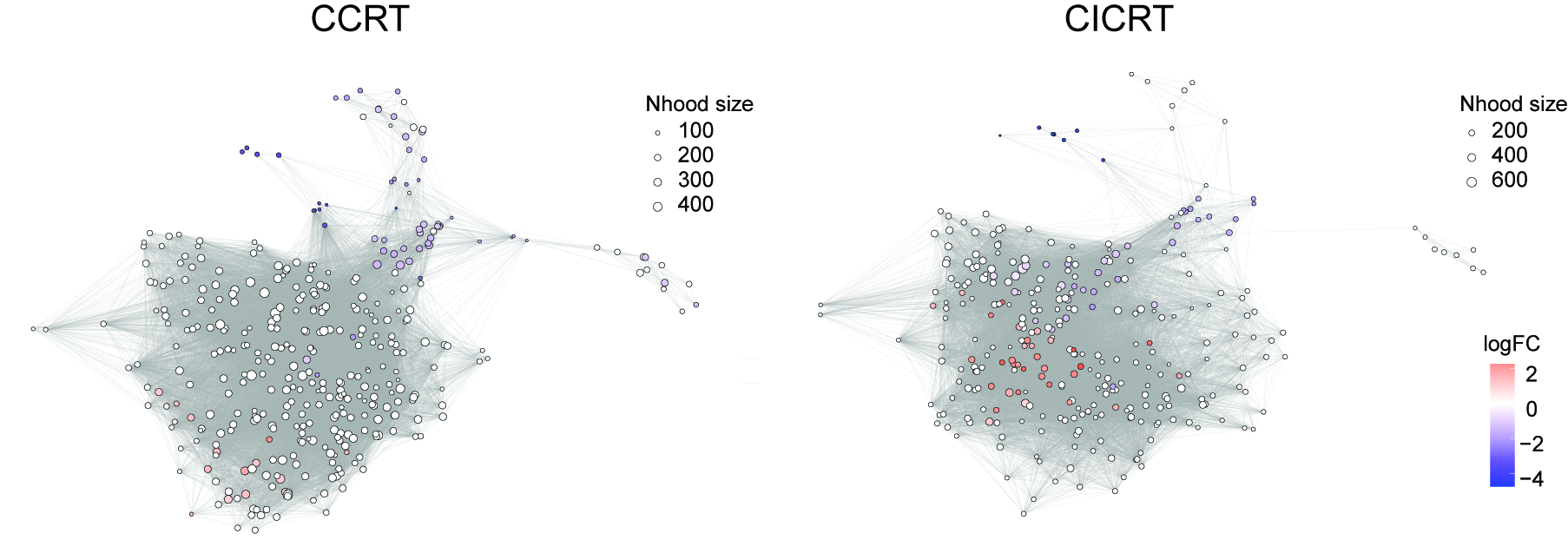


**Figure S6. Neighborhood-level differential abundance of myeloid cells following CCRT and CICRT.**

Differential abundance analysis of myeloid cell neighborhoods. Milo graphs illustrating the shift in cellular abundance between pre- and on-treatment samples for the CCRT (left) and CICRT (right) groups. Each node represents a cell neighborhood, with color intensity indicating the log2-fold change (log2FC). Red denotes enrichment in on-treatment samples, while purple denotes depletion. Node size reflects the number of cells per neighborhood (Nhood size).


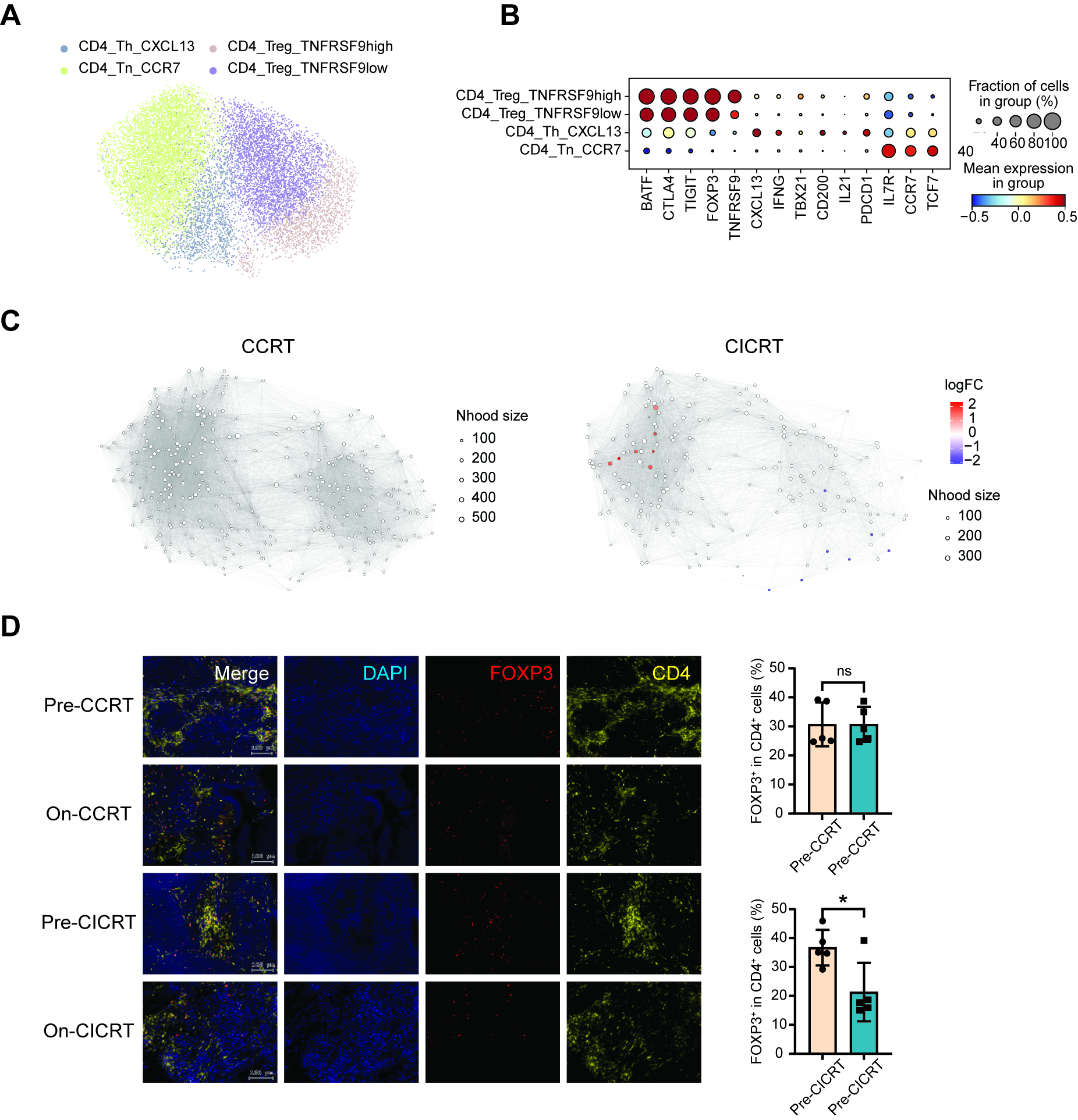


**Figure S7. Remodeling of the CD4^+^ T cell compartment following CCRT and CICRT.**

**A.** UMAP visualization of CD4^+^ T cell clusters colored by cell type.

**B.** Dot plot showing the expression of canonical marker genes across CD4^+^ T cell subclusters.

**C.** Differential abundance analysis of CD4^+^ T cells. Milo graphs showing shifts in cellular abundance between pre- and on-treatment samples for the CCRT (left) and CICRT (right) groups.

**D.** Immunofluorescence staining and quantification of CD4^+^FOXP3^+^ regulatory T cells (Tregs). Representative images showing nuclei (DAPI, blue), FOXP3^+^ cells (red), and CD4^+^ cells (yellow) in tumor tissues at pre- and on-treatment timepoints. *p*-values were determined by two-sided paired Student’s *t*-tests based on five randomly selected fields per sample.


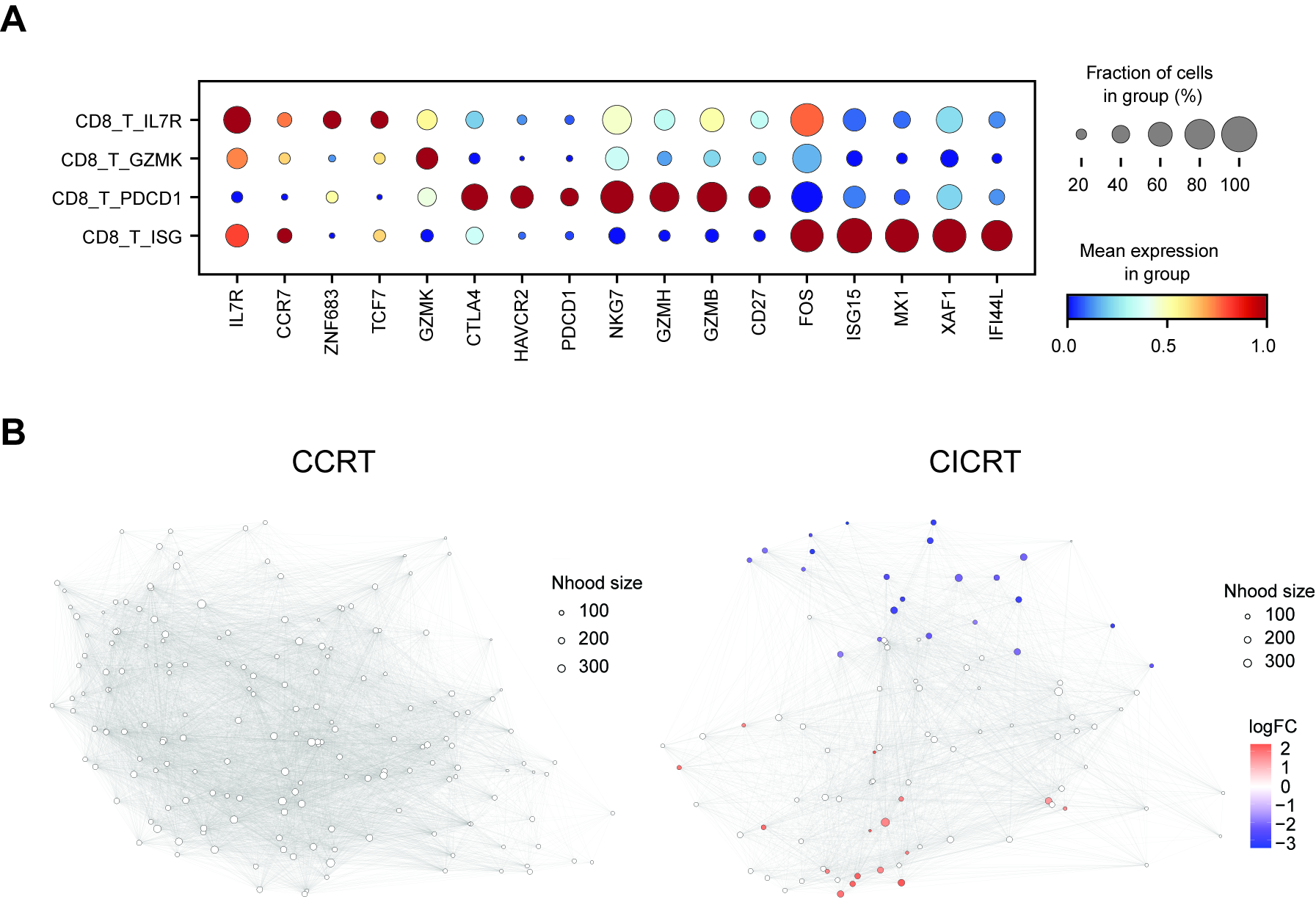
**Figure S8. Subcluster identification and differential abundance analysis of CD8^+^ T cells.**

**A.** Dot plot showing the expression of canonical marker genes in each CD8^+^ T-cell subcluster.

**B.** Differential abundance analysis of CD8^+^ T cells. Milo graphs showing shifts in cellular abundance between pre- and on-treatment samples for the CCRT (left) and CICRT (right) groups.


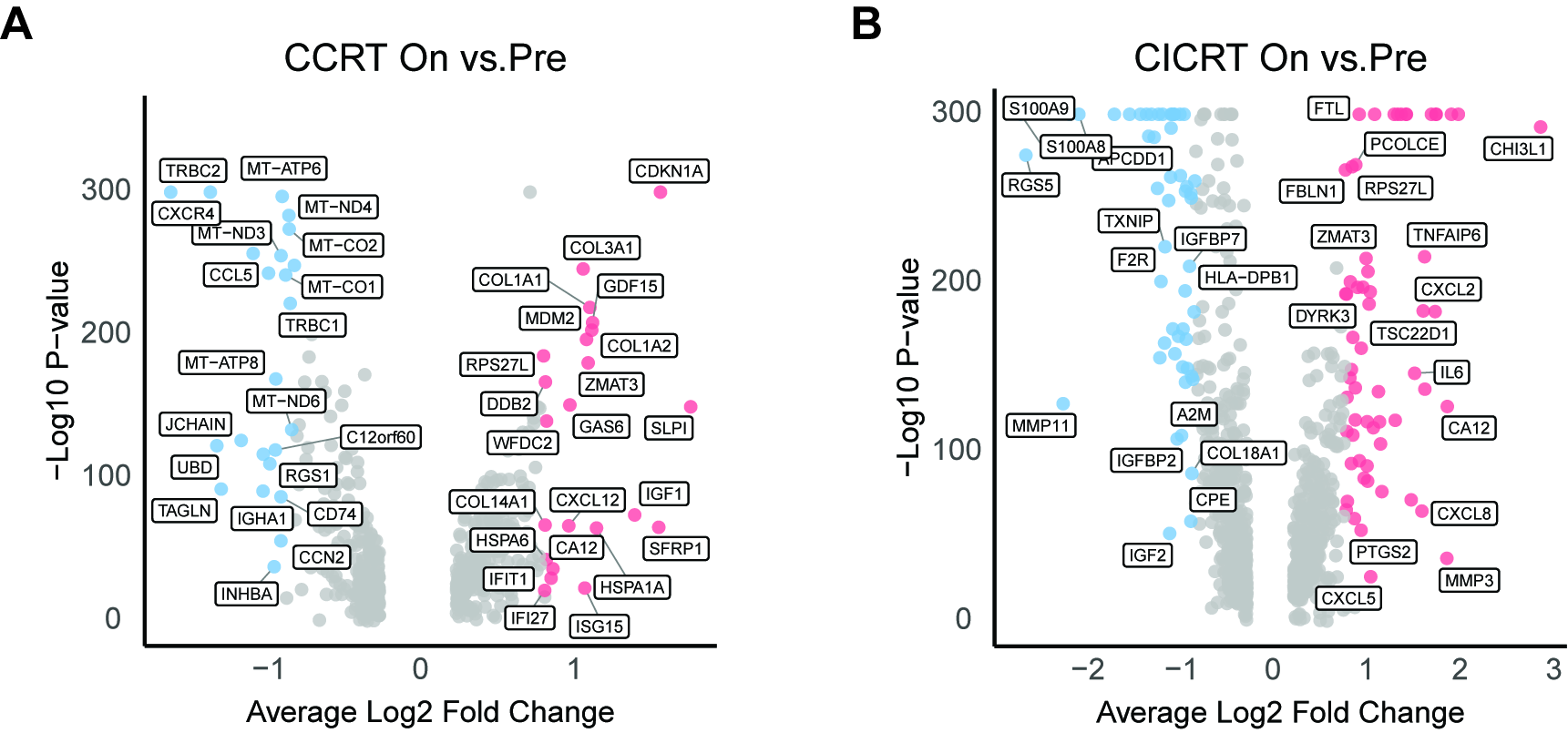


**Figure S9. Differential gene expression analysis of cancer-associated fibroblasts (CAFs) following treatment.**

**A-B.** Volcano plots depicting differential gene expression in CAFs between pre- and on-treatment samples in the CCRT (A) and CICRT (B) groups. Colored points highlight significant DEGs.

**Supplementary Table 1. Antibodies used for multiplex immunofluorescence**

| **Marker** | **Manufacturer** | **Catalog number** |
| --- | --- | --- |
| PanCK | Abcarta | PA125 |
| FOXP3 | Abcarta | PA448 |
| CD8 | Abcarta | PA577 |
| CD4 | Abcarta | PA285 |
| HLA-DR | HUABIO | ET1610-66 |
| CD163 | Abcarta | PA059 |
| CD68 | Abcarta | PA014 |
